# Complete Genome Sequencing of *Leptospira interrogans* Isolates from Malaysia Reveals Massive Genome Rearrangement but High Conservation of Virulence-Associated Genes

**DOI:** 10.3390/pathogens10091198

**Published:** 2021-09-15

**Authors:** Siti Roszilawati Ramli, Boyke Bunk, Cathrin Spröer, Robert Geffers, Michael Jarek, Sabin Bhuju, Marga Goris, Sahlawati Mustakim, Frank Pessler

**Affiliations:** 1 Research Group Biomarkers for Infectious Diseases, Helmholtz Centre for Infection Research, 38124 Braunschweig, Germany; roszilawati@moh.gov.my; 2 Department of Biotechnology, Technical University Braunschweig, 38106 Braunschweig, Germany; 3 Bacteriology Unit, Institute for Medical Research, National Institute of Health, Setia Alam 40170, Malaysia; 4 Leibniz Institute German Collection of Microorganisms and Cell Cultures (DSMZ), 38124 Braunschweig, Germany; boyke.bunk@dsmz.de (B.B.); ckc@dsmz.de (C.S.); 5 Genome Analytics, Helmholtz Centre for Infection Research, 38124 Braunschweig, Germany; robert.geffers@helmholtz-hzi.de (R.G.); Michael.Jarek@helmholtz-hzi.de (M.J.); sabinbhuju@gmail.com (S.B.); 6 Leptospirosis Reference Centre, Amsterdam Medical Centre, University of Amsterdam, 1100 DD Amsterdam, The Netherlands; m.goris@amsterdamumc.nl; 7 Department of Pathology, Hospital Tuanku Ampuan Rahimah, Klang 41672, Malaysia; sahla_17@yahoo.com; 8 Centre for Individualised Infection Medicine, 30625 Hannover, Germany; 9 Research Group Biomarkers for Infectious Diseases, TWINCORE Center for Experimental and Clinical Infection Research, 30625 Hannover, Germany

**Keywords:** biomarker, classification, genetics, genome, genome rearrangement, *Leptospira*, *Leptospira interrogans*, leptospirosis, *rfb* locus, whole genome, virulence

## Abstract

The ability of Leptospirae to persist in environments and animal hosts but to cause clinically highly variable disease in humans has made leptospirosis the most common zoonotic disease. Considering the paucity of data on variation in complete genomes of human pathogenic Leptospirae, we have used a combination of Single Molecule Real-Time (SMRT) and Illumina sequencing to obtain complete genome sequences of six human clinical *L. interrogans* isolates from Malaysia. All six contained the larger (4.28–4.56 Mb) and smaller (0.34–0.395 Mb) chromosome typical of human pathogenic Leptospirae and 0–7 plasmids. Only 24% of the plasmid sequences could be matched to databases. We identified a chromosomal core genome of 3318 coding sequences and strain-specific accessory genomes of 49–179 coding sequences. These sequences enabled detailed genomic strain typing (Genome BLAST Distance Phylogeny, DNA–DNA hybridization, and multi locus sequence typing) and phylogenetic classification (whole-genome SNP genotyping). Even though there was some shared synteny and collinearity across the six genomes, there was evidence of major genome rearrangement, likely driven by horizontal gene transfer and homologous recombination. Mobile genetic elements were identified in all strains in highly varying numbers, including in the *rfb* locus, which defines serogroups and contributes to immune escape and pathogenesis. On the other hand, there was high conservation of virulence-associated genes including those relating to sialic acid, alginate, and lipid A biosynthesis. These findings suggest (i) that the antigenic variation, adaption to various host environments, and broad spectrum of virulence of *L. interrogans* are in part due to a high degree of genomic plasticity and (ii) that human pathogenic strains maintain a core set of genes required for virulence.

## 1. Introduction

Pathogenic *Leptospira* spp. colonize the proximal renal tubules of reservoir animal hosts and are excreted through urine into the external environment. Humans acquire *Leptospira* spp. infection through contact with body fluids (e.g., urine) of carrier animals, such as rodents, and contaminated water and soils [1]. Thus, the persistence of Leptospirae in moist environments and animal hosts combined with their ability to cause clinically highly variable disease in humans has made leptospirosis one of the most common zoonotic diseases worldwide [2]. There are approximately 1.03 million cases of leptospirosis per year worldwide with a 5.7% mortality [3]. In Malaysia, human leptospirosis has been reported since 1928 and the annual incidence of leptospirosis in this country has ranged between 1 and 10 cases per 100,000 inhabitants. In 2015, 5370 cases and 30 deaths due to human leptospirosis were reported, but the true number is probably much higher because many cases, especially those with mild symptoms, are not diagnosed [4]. The genus *Leptospira* was traditionally considered to comprise approx. 20 species, but more detailed delineation by genomic techniques has recently increased this number to nearly 100 [5]. Leptospirae span a broad spectrum of virulence and hosts, ranging from low pathogenic strains in the environment to those that cause severe disease in farm animals and humans [5]. *L. interrogans* is probably the most important species causing human leptospirosis worldwide. At the same time, it can infect or colonize all mammals and was reported to be the most common *Leptospira* species among rodents in South East Asia [6]. Of the multitude of serovars and serogroups of this species, Icterohaemorrhagiae is the one most commonly associated with severe leptospirosis in humans [7].

In order to facilitate genome-based diagnostics and molecular outbreak investigations, as well as to understand the molecular evolution of phenotypic traits and genomic determinants of persistence and virulence, there have been intense efforts to elucidate the organization of the *L. interrogans* genome and variations therein among field isolates and serovars. Ren et al. described the first whole-genome sequence of *L. interrogans*, which revealed 4668 predicted genes divided between a large (4.33 Mb) and a small (359 kb) chromosome and cardinal differences from parasitic spirochetes [8]. Considering that single gene contributions to virulence are difficult to study due to the fastidious nature of Leptospirae and their resilience to genetic manipulation [9], subsequent genomic studies of Leptospirae have often focused on uncovering the genomic features underlying across-serovar differences in virulence and host adaptation. A genomic comparison of two pathogenic strains from the Icterohaemorrhagiae serogroup revealed extensive variation in the number and distribution of insertion sequences and other genomic contents, thus providing a first indication of genomic plasticity in the *L.* *interrogans* species [10]. A comparison between *L. interrogans* and the less pathogenic species *L. borgpetersenii* showed that *L. interrogans* possesses more signal transduction systems, transcriptional regulatory factors, and metabolic and solute transport functions than *L. borgpetersenii*, which may be one explanation for the remarkable ability of *L. interrogans* to adapt to various environments [11]. A subsequent genomic comparison between serovar Lai and its high-passage avirulent isolate strain IPAV uncovered a high conservation of gene contents and arrangement, but also 101 gene mutations [12]. Transfer of genes, plasmids, and other mobile genetic elements has been reported between various pathogenic Leptospirae and other bacteria; for instance, the LigA protein contains repeat regions homologous to the immunoglobulin-like regions found in the pathogenetically important intimin protein of *Escherichia coli* [13].

Major advances in our understanding of genomic diversity in the genus *Leptospira* were made in 2016. Xu et al. reported the *Leptospira* pangenome based on the draft genome sequences (i.e., not closed into contiguous sequences) of 102 global isolates, which revealed major genome variability across the genus as well as evidence of horizontal gene transfer and gene duplication during the evolution of pathogenic species [14]. In a comparative study of draft genomes of 20 *Leptospira* species, Fouts et al. identified several features of pathogenic species, including sialic acid biosynthesis, vitamin B12 autotrophy and other metabolic features, the presence of CRISPR/Cas systems, specialized protein secretion systems, and motility and chemotaxis systems [15]. Subsequent work focused on comparative studies of genome sequences from pathogenic Leptospirae from defined geographic areas. Based on the draft genomes of 22 isolates from Cuba (including 12 *L. interrogans* isolates), Noda et al. used core genome multilocus sequence typing for genomic classification (which largely agreed with conventional species assignment) and molecular epidemiological studies [16]. Santos et al. compared the draft genome sequences of 67 isolates of the *L. interrogans* serovars Copenhageni and Icterohaemorrhagiae to identify serovar-specific SNPs and indels and identified a frameshift mutation in the pic120008 gene that was specific for Icterohaemorrhagiae [17].

Taken together, these studies suggested that the high degree of inter-species genomic variability in the genus *Leptospira* extends into the species *interrogans*. However, these studies were based on draft genomes and did not feature systematic comparisons of genome organization and rearrangement based on closed genomes. We have, therefore, combined Single Molecule Real-Time (SMRT) and Illumina sequencing to obtain whole-genome sequences of six clinical *L. interrogans* isolates that were collected from a geographically defined area (the high endemic country Malaysia), and defined their cardinal shared and distinct features regarding genomic variability and potentially pathophysiologically relevant genes. Furthermore, the obtained sequences allowed an evaluation of different in silico classification schemes (Genome BLAST Distance Phylogeny, GBDP; DNA–DNA hybridization, DDH; multilocus sequence typing, MLST; and whole-genome-based SNP genotyping) for taxonomic classification of the species *L. interrogans*.

## 2. Results

### 2.1. Clinical Cases

Clinical information, including risk factors for infection, laboratory findings, and clinical severity, is summarized in Table S1. The results underscore the typical transmission of *L. interrogans* in moist environments and the broad spectrum of clinical manifestations and severity and identify 1530 as the strain associated with the mildest disease severity.

### 2.2. Classification by Serotyping

The six *L. interrogans* strains were assigned to the traditional serogroups by microagglutination test (MAT), using a panel of 43 polyclonal rabbit anti-*Leptospira* reference sera, representing 24 pathogenic and two saprophytic serogroups (Table S2). The results of the MAT and additional serotyping with monoclonal antibodies (Table S3) were not conclusive for strain 782. A cross agglutination absorption test (CAAT) was therefore performed and serovar Bindjei showed the highest similarity (Table S4). The final serogroup and serovar assignments are shown in Table 1.

### 2.3. Complete Genome Sequences

Complete closed chromosomal genome sequences could be obtained from all six strains. However, in strain 1530 the plasmids could not be resolved completely because of higher DNA fragmentation. Key features of these genomes are summarized in Table 2.

All six genomes harbored the two chromosomes typical of this species. Chromosome I contained the most substantial genome information and ranged from 4.28 to 4.56 Mb, while the smaller chromosome ranged from 350 to 395 kb. The number of plasmids ranged from 0 to 7, the number of contigs from 2 to 10. Genome size ranged from 4.6 (strain 898 and 1489) to 4.91 (782) Mb with about 3600 to 4300 CDS, but the differences were particularly due to the high number of plasmids in strain 782 and 1489 and their absence in strain 898. All strains had two 23S, two 16S, and one 5S rRNA genes. As previously described [19], the rRNA genes were not organized in operons as in most other bacteria, but were scattered across chromosome I.

### 2.4. Whole-Genome-Based Typing and Phylogenetic Classification

**Figure 1:**
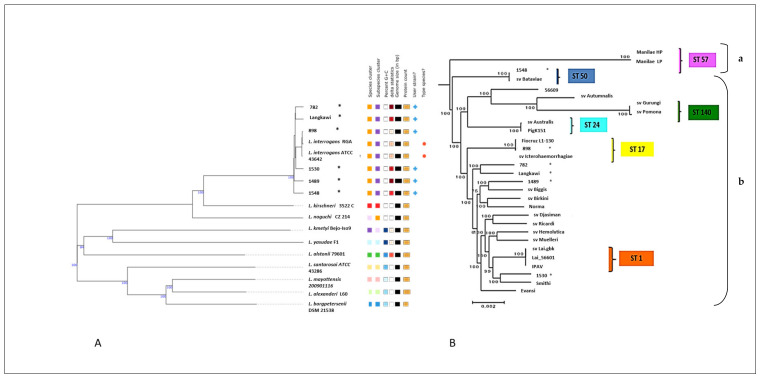
Whole genome-based phylogenetic classification of six Malaysian *L. interrogans* isolates. (**A**) Genome BLAST Distance Phylogeny (GBDP) analysis on the Type (Strain) Genome Server (TYGS) compared to two reference strains and nine non-interrogans Leptospirae. All six strains showed nearly 0.000 distance, suggesting nearly 100% homology with *L. interrogans* reference strains ATCC 43642 and RGA, clearly classifying them as belonging to this species. (**B**) Whole genome SNP genotyping. In total, using parSNP 2599 sequence clusters were generated from the six Malaysian strains plus the 23 external *L. interrogans* strains listed in Table S7, and the maximum likelihood algorithm was used to construct a phylogenetic tree. Two clades (**a,b**) are apparent. The six Malaysian strains are marked *. Sv = serovar.

We then used the whole-genome sequences for a detailed taxonomic and phylogenetic analysis. Genome BLAST Distance Phylogeny (GBDP) species typing (Figure 1A) and DNA–DNA Hybridization (DDH) (Table S5) confirmed that all strains belonged to the species *L. interrogans.* Compared with the reference genome *L. interrogans* ATCC 43642^T^ [20], strain 898 was most similar (DDH estimate 98.4%) and strain 1548 the most distant (DDH 91.3%). The findings of GBDP typing agreed well with DDH. In order to compare the genetic typing against standard serotyping in the strain collection, we then applied in silico multilocus sequence typing (MLST) based on seven housekeeping genes [21]. Results of the MLST of strain 898 and strain 1489 agreed well with the serotyping, but novel sequence types (ST) were assigned to four other strains: Langkawi (ST236), 1530 (ST237), 782 (ST240), and 1489 (ST241). New alleles for the *pntA* and *pfkB* genes were identified in Langkawi and 1530, respectively (Table S6).

We then performed whole-genome SNP genotyping to analyze the inter-strain relationships of the 29 *L. interrogans* strains, comprising the 6 and 15 Malaysian strains whose complete and draft genomes, respectively (listed in Table S7), were obtained in this study, and eight publicly available *L. interrogans* complete genome sequences (AE010300.2, NC_005823.1, NZ_CP012603.1, NC_017551.1, NZ_CP013147.1, NZ_CP011931.1, NZ_CP011934.1, NZ_CP011410.1). Core genome alignment using Parsnp was applied to these 29 *L. interrogans* strains. It created 2599 clusters, with an average of 16 maximal unique matches (MUMs) per cluster (average cluster length 1327 bp), corresponding to a 75.5% coverage of the reference genome, i.e., serovar Lai, strain 56601. A phylogenetic tree was generated using the identified core genome SNPs (*n* = 2599) and the maximum likelihood method (Figure 1B), which revealed two major *L. interrogans* lineages, with a smaller clade A and a larger clade B containing six nested subclades. Comparing the results obtained with these two molecular typing methods, we found that MLST agreed well with the SNP genotyping, as strains with similar STs (i.e., ST1, ST17, ST24, ST50, ST57, and ST140) were grouped in the same cluster in the whole genome SNP tree. We also noted that strain 898 is closely related to serovar Icterohaemorrhagiae (length difference 0.00%). Based on Malaysian epidemiological data, this is quite plausible as Icterohaemorrhagiae is a common circulating serovar in Malaysia [22]. Although strains Langkawi and 782 were phylogenetically the most closely related, they were isolated 10 years apart from each other and in different locations. Moreover, even though five of our strains were isolated during one continuous 18-month period, the above phylogenomic analysis suggests that they originated from diverse sources. Taken together, the above results underscore the usefulness of genome-based classification methods to understand the taxonomic relationships within the species *L. interrogans* in a high endemicity country like Malaysia.

### 2.5. Genome Organization and Rearrangements

**Figure 2:**
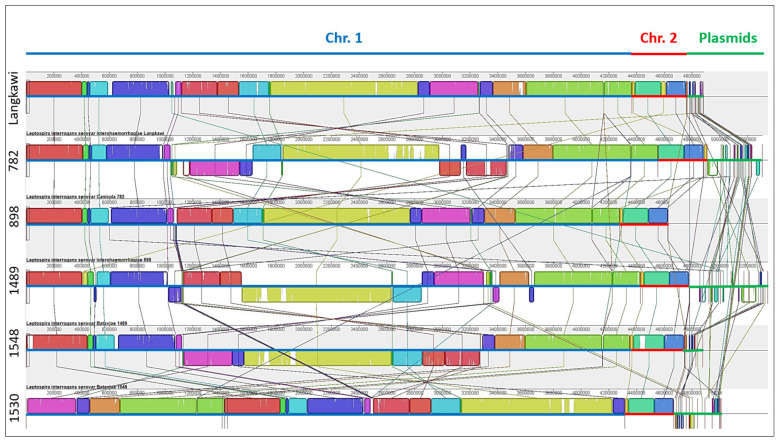
Genome synteny of six Malaysian *L. interrogans* strains revealing substantial genome rearrangement. The strains were aligned and arranged using Mauve genome aligner in the following order: (top) Langkawi, 782, 898, 1489, 1548, 1530 (bottom). Chromosome I is shown as the first element per genome and is highlighted by a blue line, the much more conserved chromosome II is highlighted by a red line, and additionally occurring plasmids by a green center line. LCB correspond mainly to conserved synteny regions, as represented by colored boxes. The lines between the genomes connect the blocks that are conserved between two strains, indicating rearrangements. The genome of strain 1530 (last line) was recorded as a draft genome and thus not rotated to the *dnaA* gene so that the starting position of chromosome I differs in comparison to the others. The horizontal colored lines beneath each block indicate chromosome I (blue), chromosome II (red), and plasmids (green).

**Figure 3:**
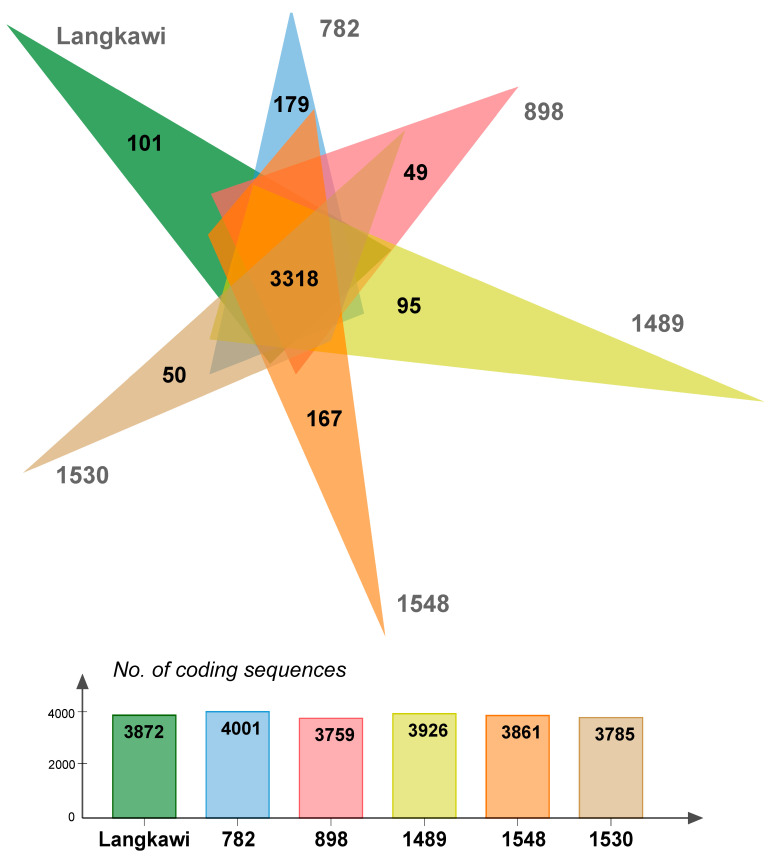
Core and accessory genomes based on the chromosomal CDS of six Malaysian *L. interrogans* strains. Proteinortho analysis was performed using the sequences of chromosomes I and II as input. The Venn diagram identifies the chromosomal core genome of 3318 CDS in the center intersect. The number of strain-specific CDS, ranging from 49 to 179, is indicated in the diagram rays. The number of CDS shared with 1–4 of the other strains is as follows: strain Langkawi, 19; strain 782, 504; strain 898, 392; strain 1489, 513; strain 1548, 376; and strain 1530, 417. The bottom panel states the number of chromosomal coding sequences for each strain.

In order to visualize the overall genome organization and to uncover potential genome rearrangements across strains, conserved syntenic regions were visualized using Mauve genome aligner (Figure 2). Large collinear blocks (LCBs) are identified by differently colored boxes and strain-specific regions (genomic islands, GI) either by white regions within LCBs or by the spaces in between. Although both chromosomes could be found in all strains, only chromosome II was highly conserved across all strains. The sizes and positioning of the central LCBs within chromosome I differed substantially, indicating genomic islands and major genomic rearrangements. Insertion sequence (IS) elements are often located at the junctions of those rearrangements, which might lead to recombination events. Only weak similarities between plasmids were detected. Strain 1530, which was associated with the mildest clinical disease (Table S1), also displayed a genome arrangement that differed from the other strains. Next, we determined the core and accessory (unique) genomes of the six strains. Proteinortho analysis identified a core genome of 3271 CDS, and an accessory genome (i.e., the sum of CDS not shared by all six strains) of 2150 CDS (Figure 3). The size of the accessory genome unique to each strain varied from 49 (strain 898) to 179 CDS (strain 782).

### 2.6. Arrangement of the rfb Locus

**Figure 4:**
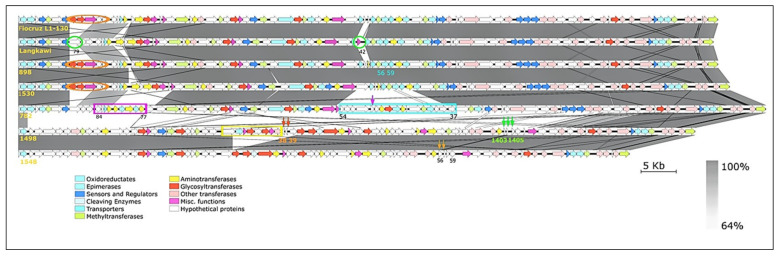
The *rfb* locus of six Malaysian *L. interrogans* isolates with reference to *L. interrogans* serovar Copenhageni strain Fiocruz L1-130. The *rfb* locus is located between the *marR* (transcriptional regulator) and *sdcS* (Na^+^/dicarboxylate symporter) genes. The largest *rfb* locus is present in 782, which is due to the presence of two large additional gene clusters. Interestingly, the arrangement (and gene content) of strains 1498 and 548 is quite different from the other four genomes, but within themselves they are quite similarly organized. Generally, strain Langkawi is similarly organized as the serogroup Icterohaemorrhagiae strains (898, 1530, Fiocruz L1-130); however, it lacks a series of genes present in other Icterohaemorrhagiae strains (marked by red circles) and has two additional genes (green circles). Additional gene clusters within strain 782 are marked by blue and pink rectangles and in 1489 by a yellow rectangle. The inserted transposases within two clusters in 1489 are indicated by red and green arrows, those within one cluster in 1548 are indicated by orange arrows, and one transposase in 782 is indicated by a pink arrow.

The *rfb* locus encodes the O-antigen, which is the basis for *Leptospira* serovar identification and also contributes to pathogenicity [15]. Serovar classification of leptospires is based on expression of the epitopes arranged in a mosaic pattern on the surface of the LPS layer [23]. We compared the *rfb* loci of the six strains against Fiocruz L1-130 as the reference genome (Figure 4). The crude pattern and arrangement of the gene clusters of O-antigen correlated with the serotyping results; i.e., Fiocruz, strain 898, strain 1530, and Langkawi were assigned to serogroup Icterohaemorrhagiae, and strain 1489 and strain 1548 to serogroup Bataviae. Strain 782 was classified belonging to the Canicola serogroup. Strain 898 and strain 1530 were similarly organized. A gene arrangement analysis of the six strains according to the other functional classes revealed that the gene arrangement and content of strains 1498 and 1548 are closely related but differ from the others. Strain Langkawi displays a gene combination unique from 782 (orange circle), 1530 (green circle), as well as a galactoside O-acetyltransferase (blue circle), which sets it apart from the other Icterohaemorrhagiae serogroup strains. Strains Fiocruz, 898, and 1530 have similar gene arrangements. Interestingly, transposase gene fragments are present in the *rfb* locus of 782, 1489, and 1548 (the non-Icterohaemorrhagiae serogroup) but not within the three Icterohaemorrhagiae strains (Figure 4). In all three strains, transposases within the *rfb* locus were found to be frameshifted (locus tags Lepto782_12890, Lepto1489_07680, Lepto1489_07670-80, and Lepto1548_07880-85) and therefore most presumably inactive.

### 2.7. Mobile Genetic Elements

*Phages and CRISPR systems.* In silico phage finding suggested that the six strains contained between 1 and 10 phages (Table S8). However, none of them were found to be complete, suggesting that they represent remnants of earlier infections that were controlled by the CRISPR systems predicted to be fully functional, which were found in all six genomes (Table S9). Specifically, there were 10 to 16 loci with 1 to 13 spacers in the CRISPR arrays in each of the six genomes. Strain 1548 had the most (6 arrays) and 782, 898, and 1489 the fewest (2 arrays) confirmed CRISPR arrays. The most frequently found spacer element originated from plasmid *lcp1* from *L. interrogans* serovar Linhai, followed by plasmid *lcp3* from the same serovar [24].

*Plasmids.* Contigs not belonging to chromosome I and II were designated as plasmids. Plasmid sizes range from 51 to 101 kb. Even though up to seven plasmids could be identified, only up to 24% of their sequences could be matched with sequences in NCBI GenBank and assigned a functional annotation (Table S10). The predicted plasmid CDS were functionally annotated using eggNOG, where the assigned orthologous groups were most frequently associated with replication, recombination, and repair (Figure S1). Most of the plasmids could be identified as conjugative by identification of a parB-like plasmid partitioning system (Table S10).

*Insertion Sequences (IS)*. Pathogenic Leptospirae possess more than 20 types of IS in their genome and IS are considered among the driving forces of leptospiral genome evolution and diversification [15]. A detailed comparison of IS in the six strains is shown in Table S11. IS were detected both on chromosomes and plasmids. Strain 1489 had the highest number of IS followed by strains 1548 and 1530. The majority of IS belonged to IS*Lin*1 followed by IS*Lin*2, both being located on chromosome I.

### 2.8. Genes Associated with Virulence

We compared amino acid sequences of genes involved in synthesis of lipid A, a component of the LPS layer that has different acyl chains and phosphorylation sites that can abrogate endotoxinogenicity [25]. The six strains could be differentiated by the 3-deoxy-manno-octulosonate cytidylyltransferase gene (*kdsB1*)*,* which divided the strains into two groups based on their similarity to serovar Copenhageni (Fiocruz L1-130) and the Icterohaemorrhagiae serogroup: strain Langkawi, strain 898, and strain 1530, all of which belong to serogroup Icterohaemorrhagiae, had high similarity, whereas strains 782, 1489, and 1548 had low similarity. The sequences of the other 12 enzymes involved in lipid A biosynthesis were 99–100% identical to each other (Table S12). Although its mechanistic implications for the post-translational protein modifications of Leptospirae remain incompletely understood, the ability to synthesize sialic acid has been detected in pathogenic Leptospirae [15,26]. All six strains contained 13 key genes responsible for sialic acid synthesis (Table S13). The degree of homology classified the strains into two groups: four strains were highly homologous (99–100%) to the reference strain *L. interrogans* serovar Copenhageni Fiocruz L1-130 in all 13 proteins, whereas strains 1489 and 1548 had markedly lower homologies (39–93%) in all targets except UDP-N-acetylglucosamine diphosphorylase.

### 2.9. Alginate Biosynthesis and Other Virulence Factors

The alginate biosynthesis pathway is important for bacterial biofilm formation [27]. All six *L. interrogans* genomes contained up to 10 copies of scattered *algl* (alginate O-acetylaselyase) in chromosome I homologous to *Pseudomonas aeruginosa (P. aeruginosa)* PAO1 (ranging 90–99%), suggesting that these strains could produce alginate-containing biofilms. In order to test how extensive the homology was across the entire pathway, we queried for homologies with all *P. aeruginosa* proteins potentially involved in alginate biosynthesis and transport, as well as the factors involved in phenotypic/regulatory switching in this pathway (Table S14). Genes encoding the inner membrane and periplasmic proteins were found in all six genomes, but were scattered across chromosome I and not arranged in a single gene cluster as in *P. aeruginosa*. In addition, there were no homologs of the *P. aeruginosa* alginate export proteins (AlgK and AlgE), and high conservation only for one regulatory (AlgB) and one phenotypic switching (MucD) factor [28]. Thus, even though key mediators of alginate biosynthesis were detected across all six strains, there likely are important differences from *P. aeruginosa* in terms of extracellular export and phenotypic regulation (illustrated in Figure S2). Murray proposed that thirteen experimentally validated virulence factors are found in *L. interrogans* [29]. All of them were nearly 100% conserved in all six strains, except la_1641 (only 100% conserved in serovar group Icterohaemorrhagiae and Canicola but not in Bataviae) and lman_1408. Both proteins are involved in LPS synthesis.

### 2.10. Potential Virulence Factors by Homology Search against Other Prokaryotes

In order to identify additional potential virulence factors, we used the Virulence Factor Database (VFDB) as reference under the assumption that a virulence factor that has been validated in other pathogenic bacteria is likely relevant to pathogenic leptospires. Among other potential virulence factors, this analysis identified enzymes involved in membrane signaling, tissue degradation, complement inactivation, oxidative stress responses, antiphagocytic capsule, efflux pump, chemotaxin proteins, and type II and III secretion systems (Table S15).

### 2.11. Antibiotic Resistance Genes

A search for CDS that are potentially associated with antimicrobial resistance did not reveal significant homologies with any plasmid-encoded or chromosomal resistance genes known from other bacteria.

## 3. Discussion

Considering the persisting challenges in taxonomic classification, diagnosis, and genotype–phenotype correlations in pathogenic Leptospirae, we sequenced and acquired the complete genome sequences of six *L. interrogans* strains from Malaysia, a country with high endemicity of this pathogen, enabling the mapping of a definitive genome structure and subsequent detailed genome-wide comparisons.

We found, on one hand, considerable shared synteny in gene organization and contents, but also evidence of massive genomic rearrangement and plasticity, as well as greatly varying strain-specific and overlapping (shared by 2–5 strains) accessory genomes. These seemingly contradictory findings are best explained by the need of the pathogen to maintain a core genome that mediates the functions for persistence and transmission in a common “envirome”, but at the same time to provide enough responsiveness in the genome to allow for adaptation in the face of environmental and immune-mediated pressure. The potential for genomic flexibility is manifested in these strains primarily by the wide occurrence of mobile elements, i.e., plasmids, insertion sequences, and prophages, which were found in all six strains but varied highly in number and distribution throughout the genome. It has been suggested that such mobile elements support ongoing genome plasticity by homologous recombination and horizontal gene transfer [30]. It is worth noting that we found transposases and clear evidence of rearrangement in the *rfb* locus, which is the key antigen in host immunity to leptospirosis and the classic antigen used for serotyping. The number of plasmids varied highly among the six strains. Intriguingly, only a minority of plasmid-encoded sequences could be matched with known sequences, and it is tempting to speculate that some of the remaining sequences contribute to adaptation and virulence by virtue of potentially novel functions.

Previous studies have shown that prophages occur in *Leptospira* genomes [31] and that they are more common in pathogenic and intermediate leptospires [15]. Prophages were found in all six strains in highly varying numbers and locations, underscoring the plasticity of these genomes. Interestingly, all of them were found to be incomplete, most likely as a consequence of active CRISPR/Cas systems in all strains. Fouts et al. suggested that only pathogenic Leptospirae possess functioning CRISPR systems [15]. In our study, the number of CRISPR arrays varied highly across the strains, and future work should address whether their number and/or arrangement contributes to pathogenicity or environmental adaptation [15].

We found considerable conservation of loci previously implicated in *L. interrogans* virulence, including 100% amino acid sequence identity in all 13 factors that had been defined by Murray et al. as experimentally validated in acute infection [29]. In addition, high conservation was found in all key enzymes of the lipid A pathway, and in those components of the alginate pathway (which is important for the formation of biofilms) that were detected in the six genomes; even though there was more variability in the components governing sialic acid synthesis, all key components of the pathway were detected in all six strains. The homology search against known virulence factors from other bacteria revealed at least the possibility that *L. interrogans* encodes a much broader range of virulence factors than previously thought, which opens up exciting opportunities to identify additional determinants of clinical severity in humans as well as potential treatment targets. Interestingly, we found strong homologies to the antiphagocytic cap8EFG protein from *S. aureus* in the O-antigen locus [32], whereas in *L. interrogans* serogroup Bataviae a sequence homologous to complement-inactivating protein NeuBC from *Streptococcus agalactiae* is found in this locus, further underscoring the importance of plasticity within this locus [33]. Another potential defensive mechanism found was a homology to the MtrD efflux pump of *Neisseria gonorrhea*. Its presence in four copies in all strains indicates that it is one of the essential defensive strategies used by *L. interrogans* [34], for instance by supporting growth under hostile conditions encountered in vivo.

As opposed to some Gram-negative bacteria, which possess six or more types of secretion systems, only the Type 1 (T1SS) and Type 2 (T2SS) secretion systems have been reported in Leptospirae [9]. Although a pathogenic role of T2SS in Leptospirae has not been demonstrated, several components of the T2SS are encoded in the leptospiral genome, and we found several homologies (e-value higher than 10^−52^) with T2SS from other pathogenic bacteria in all six strains, i.e., GspD and GspE from *Shigella dysenteriae* and XcpS from *P. aeruginosa*, the latter of which is required for the translocation of a variety of toxins and enzymes across the outer membrane into extracellular fluid [35,36]. It is still a mystery how Leptospirae invade host cells without having the T3SS similar to some obligate and facultative intracellular bacteria. T3SS is critical in some pathogens for invasion and survival in host cells. Interestingly, we found a significant sequence homology to the CdsN protein, which acts as an ATPase for T3SS in *Chlamydia trachomatis,* in all six *L. interrogans* strains with a conserved locus tag. In *C. trachomatis*, CdsN is an ATPase that catalyzes the unfolding of proteins and the secretion of effector proteins through the injectisome and interacts with a putative chaperone, Cpn0706, and the putative plug and effector protein. However, we did not find homologies with Cpn0706 or CopN in *L. interrogans*, and the function of CdsN as a T3SS in *L. interrogans* therefore remains doubtful [37].

Consistent with the nearly universal susceptibility of Leptospirae to penicillins, we did not detect any plasmid-encoded antibiotic resistance genes such as beta-lactamases. Likewise, there were no acquired mutations associated with antimicrobial resistance in other bacterial pathogens. These results support clinical experience that antibiotic resistance is still not an emerging problem in *L. interrogans*; however, these findings may change as more isolates are examined [38].

## 4. Materials and Methods

### 4.1. Culture Collection

In total, 2000 blood samples from patients suspected of leptospirosis admitted to Hospital Tuanku Ampuan Rahimah (HTAR), Klang, Selangor (Malaysia), were collected from 1 January 2014 until 31 December 2015. Samples were cultured in modified Ellinghausen–McCullough–Johnson–Harris (EMJH) media and incubated at 30 °C for four weeks, as described [39]. *L. interrogans* isolates 782, 898, 1489, 1548, and 1530 were isolated from five patients admitted to HTAR with a clinical diagnosis of leptospirosis. Clinical and laboratory data of the five patients were recorded. One isolate originating from Langkawi, Malaysia [18], was purchased from the Leptospirosis Reference Centre, Amsterdam.

### 4.2. DNA Extraction

For DNA extraction, a 7–10-day-old *L. interrogans* culture in 30 mL of EMJH medium was centrifuged and the pellet was resuspended in 180 μL Buffer ATL (Qiagen, Hilden, Germany). Genomic DNA extraction from the isolates was performed using the QIAamp DNA Mini Kit. The extracted DNA was stored at −70 °C until sequencing.

### 4.3. PacBio Library Preparation and Sequencing

The SMRTbell™ template library was prepared according to the instructions from Pacific Biosciences (Menlo Park, CA, USA) following the Procedure & Checklist—Greater Than 10 kb Template Preparation. To prepare 15 kb libraries, 8 µg genomic DNA was sheared using g-tubes™ from Covaris (Woburn, MA, USA). DNA was end-repaired and ligated overnight to hairpin adapters applying components from the DNA/Polymerase Binding Kit P6 (Pacific Biosciences). BluePippin™ Size-Selection (Sage Science, Beverly, MA, USA) to greater than 4 kb was performed according to the manufacturer’s instructions. Conditions for annealing of sequencing primers and binding of polymerase to purified SMRTbell™ template were assessed with the Calculator in RS Remote (Pacific Biosciences). SMRT sequencing was carried out on the PacBio RSII (Pacific Biosciences) taking one 240-min movie for one to three SMRT cells per isolate using P6 Chemistry.

### 4.4. Illumina Library Preparation and Sequencing

For short reads, libraries for whole genome sequencing were prepared with the NEBNext^®^ Ultra™ DNA Library Prep Kit for Illumina^®^ with 550 bp as insert size. Sequencing using the MiSeq Personal Sequencer (Illumina Inc., San Diego, CA, USA) was done to 250 cycles in both directions. The generated sequencing reads were de novo assembled with the VELVET tool version 1.2.10. (www.ebi.ac.uk/~zerbino/velvet/ (accessed on 29 March 2017)) [40].

### 4.5. Genome Assembly and Annotation

SMRT Cell data were assembled using the “RS_HGAP_Assembly.3” protocol included in SMRT Portal version 2.3.0 using default parameters. The assemblies revealed circular chromosomes and plasmids. All replicons were circularized, and particularly the artificial redundancies at the ends of the contigs were removed and adjusted to *dnaA* or the respective plasmid replication gene as the first gene. Error correction was performed by mapping of Illumina MiSeq data onto finished genomes using BWA 1.1.4 (http://bio-bwa.sourceforge.net/ (accessed on 29 March 2017)) [41], with the subsequent variant and consensus calling using VarScan 2 (http://varscan.sourceforge.net/ (accessed on 29 March 2017)) [42]. A consensus concordance of QV60 could be confirmed for all genomes. Draft annotation was carried out using Prokka 1.8 (https://kbase.us/applist/apps/ProkkaAnnotation.net/ (accessed on 29 March 2017)) [43]. The genome sequences were deposited at NCBI GenBank annotated by the NCBI Prokaryotic Genome annotation Pipeline (https://www.ncbi.nlm.nih.gov/genome/annotation_prok/ (accessed on 29 March 2017)) under Accession Numbers CP043876-CP043901 and VWNG00000000, respectively.

### 4.6. Additional L. interrogans Genome Sequences

Seven additional complete genomes of *L. interrogans* were obtained from NCBI RefSeq and used as query genomes. In addition, we obtained 15 previously unsequenced Malaysian strains from Leptospirosis Reference Centre, Amsterdam, and prepared draft genomes by Illumina sequencing.

### 4.7. Serotyping

Typing with polyclonal reference sera (using microagglutination test i.e., MAT) and monoclonal antibodies to identify serogroups and serovars was performed by the WHO Leptospirosis Reference Centre, Amsterdam, as described before [44].

### 4.8. Cross Agglutination Absorption Test

The CAAT protocol was described in [45]. First, the results of cross agglutination between rabbit antiserum of serovars from serogroup Canicola with the antigen of interest, i.e., strain 782, were assessed. Antigen–antiserum combinations with heterologous agglutination (50%) were later subjected to absorption tests using antisera and antigens (live and dead) from respective serovars from the Canicola serogroup. The serovar with maximum absorption (least agglutination) was considered the homologous serovar.

### 4.9. In Silico DNA Hybridization

Genome-to-Genome Distance Calculator (GGDC) is an in silico tool for genome-to-genome comparison that mimicks conventional DDH [46]. The DNA G + C content was calculated from the genome sequences. The resulting G + C content differences were compared with DDH similarities calculated in silico using the GGDC web server (http://ggdc.dsmz.de/ (accessed on 13 May 2017)), with 70% similarity as the gold standard threshold for species boundaries. The results indicated that the G + C content, if computed from genome sequences, varied no more than 1% within species. The sequence of the reference strain, *L. interrogans* ATCC 43642^T^, was downloaded from https://www.ncbi.nlm.nih.gov/assembly/GCF_900156205.1 (accessed on 13 May 2017).

### 4.10. Genome BLAST Distance Phylogeny (GBDP) Species Typing

The Type (Strain) Genome Server (TYGS, https://tygs.dsmz.de (accessed on 13 May 2017)) was used to infer a phylogenetic tree with FastME 2.1.6.1 from GBDP distances calculated from genome sequences. The branch lengths are scaled in terms of GBDP distance formula *d*_5_. The numbers above the branches are GBDP pseudo-bootstrap support values > 60% from 100 replicates, with an average branch support of 61.4%. The tree was rooted at the midpoint analyses [47].

### 4.11. Multilocus Sequence Typing (MLST)

The nucleotide sequences of seven MLST housekeeping genes (*glmU*, *pntA*, *sucA*, *tpiA*, *pfkB*, *mreA* and *caiB*) were extracted from all 29 genomes, and sequence types (STs) assigned using the MLST website (http://leptospira.mlst. net/ (accessed on 20 April 2017)) [21].

### 4.12. Single Nucleotide Polymorphism (SNP) Phylogeny Analysis

Genome-wide single nucleotide polymorphism (SNP) patterns were analyzed with ParSNP v1.2 (https://github.com/marbl/parsnp (accessed on 13 May 2017)) [48], a core genome aligner that focuses on identifying the set of orthologous sequences conserved in all aligned genomes. FASTA files of the six genomes of interest were used as input, and whole-genome alignment and mapping were performed against the first chromosome of the *L. interrogans* serovar Lai strain 56601 (RefSeq NC_004342.2). Within ParSNP, FastTree2 was used to compute a SNP tree, which was computed using the maximum likelihood algorithm with 1000 permutations. Output was produced as multi-alignments (XMFA), variants (VCF), core genome phylogeny (Newick), and Gingr input format (GGR). The Newick files were later exported into MEGA 7 [49], for obtaining a better resolution of the phylogenetic trees.

### 4.13. Core Genome Analysis

The following general parameters of five complete genomes and one draft genome were documented: size of chromosome I and II; number of contigs in each genome; and number of tmRNA, rRNA, tRNA, repeat regions, genes, coding sequences (CDS), signal peptides, and miscellaneous RNA.

### 4.14. Protein Orthology

Clusters of orthologous proteins were generated using ProteinOrtho V5.16b (https://www.bioinf.uni-leipzig.de/Software/proteinortho/ (accessed on 23 May 2017)) [50]. The core genome was analyzed based on the number of common proteins present in all six strains. Paralogous proteins were gathered in the same clusters and unique genes were calculated for each strain applying the—singles option. Orthologous proteins were visualized in a Venn diagram (http://jvenn.toulouse.inra.fr (accessed on 17 August 2021)) [51].

### 4.15. Genome Synteny

An analysis for genome synteny applying LCB was performed using Mauve 2.3.1 2.4 (https://www.mybiosoftware.com/mauve-2-3-1-multiple-genome-alignments.html (accessed on 25 May 2017)) [52].

### 4.16. Plasmids

Contigs that did not belong to chromosome I and II were considered plasmids. The fasta files of the contigs were queried in Blastn for identification of the plasmids. Orthologous Group (OG)-based functional annotation of CDS predicted in each plasmid was performed using eggNOG 5.0 (http://eggnog5.embl.de (accessed on 25 September 2017)) [53].

### 4.17. Prophages

Genomes were queried for the presence of phages using PHASTER (PHAge Search Tool Enhanced Release) at (http://phaster.ca (accessed on 25 September 2017)) [54].

### 4.18. Clustered Regularly Interspaced Short Palindromic Repeats (CRISPR)

Genome sequences were queried for Clustered Regularly Interspaced Short Pallindromic Repeats (CRISPR) elements using CRISPRCasFinder (https://crisprcas.i2bc.paris-saclay.fr/ (accessed on 25 September 2017)) [55]. Spacers of CRISPR arrays were checked for the presence of mobile genetic elements by the blastn search engine [56].

### 4.19. Insertion Sequences (IS)

Genome sequences were compared against IS database using ISFinder (https://isfinder.biotoul.fr/ (accessed on 25 May 2018)) [57]. Only hits of equal to expectation value (e-value) 0.0 were included. Transposases of the *rfb* locus were queried by Blastp search [56].

### 4.20. Antimicrobial Resistance Gene

The genomes were queried for chromosomal mutations and acquired genes associated with antimicrobial resistance genes using ResFinder 3.2 (https://cge.cbs.dtu.dk/services/ResFinder/ (accessed on 27 May 2018)) [58].

### 4.21. O-Antigen Analysis

The LPS biosynthetic system (*rfb* locus) of all six strains was compared using EasyFig [59] and color-coded similarly to Fouts et al. [15]. Amino acid sequences of each genome corresponding to lipid A and sialic acid biosynthesis, as studied in [15], were queried against those of *L. interrogans* serovar Copenhageni strain Fiocruz L1-130 using Blastp search (https://blast.ncbi.nlm.nih.gov (accessed on 30 May 2018)) [56]. Locus tags and e-values were recorded.

### 4.22. Bacterial Virulence Factors

Protein sequences of each genome were queried against 2602 sequences of proteins associated with experimentally verified virulence factors from other bacteria in (VFDB) (http://www.mgc.ac.cn (accessed on 15 September 2021)) [60]. Hits with e-value ≤ 1 × 10^−52^, which represents the highest score of alignment, i.e., ≥200, were considered significant.

### 4.23. Alginate Biosynthesis

Proteins associated with structural biosynthesis, regulation, and genotypic switching relating to alginate biosynthesis in the *P. aeruginosa* PAO1 reference strain were compared against the six *L. interrogans* genomes using Blastp search [27].

## 5. Conclusions

*Leptospira interrogans* is a highly dynamic pathogen that can survive in multiple hosts and environments and causes a broad spectrum of clinical severity in humans. Our study underscores the variability and plasticity of its genome, but also highlights conserved features that may be critical for pathogenesis in humans. Future studies should aim to unravel the relative contributions of the conserved and adaptive features of this pathogen to outbreak dynamics and pathogenicity.

## Figures and Tables

**Table 1:** Serogroup and serovar assignment to six Malaysian *L. interrogans* strains ^§^.

Strain	Serogroup	Serovar
Langkawi *	Icterohaemorrhagiae	Lai-like
898	Icterohaemorrhagiae	Copenhageni/Icterohaemorrhagiae
782	Canicola	Bindjei
1489	Bataviae	Paidjan
1548	Bataviae	Bataviae
1530	Icterohaemorrhagiae	Yeoncheon

^§^ Summary based on the serotyping results listed in Tables S2 and S3, except for serovar assignment of strain 782, was obtained by a cross agglutinin absorption test. * Based on previous publication [18].

**Table 2:** Genome properties of the *L. interrogans* strains investigated in this study based on Prokka annotations.

Feature		Strain				
	Langkawi	782	898	1489	1548	1530
Size (Mb)	4.76	4.91	4.63	4.63	4.74	4.67
Chromosome I (Mb)	4.37	4.56	4.28	4.28	4.35	4.32
Chromosome II (Mb)	0.39	0.35	0.35	0.35	0.39	0.35
No. of plasmids	2	5	0	7	2	≥5 *
No. of contigs	4	7	2	9	4	10
CDS	3872	4001	3759	3926	3861	3785
tmRNA	1	1	1	1	1	1
Repeat region ^	1	2	1	2	3	2
Gene	4048	4476	3716	4516	4000	4087
tRNA	37	37	37	37	37	37
23S	2	2	2	2	2	2
16S	2	2	2	2	2	2
5S	1	1	1	1	1	1
Signal peptide	132	129	124	133	131	125
Miscellaneous RNA	5	11	5	10	5	0

* The plasmids could not be resolved completely in strain 1530 because of higher DNA fragmentation. ^ Referring to CRISPR repeats only. CDS, coding sequence; tRNA, transfer ribonucleic acid; tmRNA, transfer-messenger ribonucleic acid.

## Data Availability

All genome sequences were deposited at NCBI under Accession Numbers CP043876-CP043901 and VWNG00000000, respectively. The corresponding BioProject Accession Numbers are PRJNA564735 (LangKawi), PRJNA564736 (1548), PRJNA564737 (782), PRJNA564738 (898), PRJNA564739 (1498) and PRJNA564740 (1530).

## Supplementary Materials

The following are available online at https://www.mdpi.com/article/10.3390/pathogens10091198/s1. Figure S1: Functional annotation of proteins in the plasmids of the five plasmid-containing *L. interrogans* strains, Figure S2: Model of alginate biosynthesis, regulatory and genotypic switching of *L. interrogans* in reference to proteins involved in alginate synthesis in *P. aeruginosa* PAO1, Table S1: Summary of sociodemographic and clinical data of six patients with *L. interrogans* infection contracted in Malaysia, Table S2: Summary of serogroup typing by microscopic agglutination test with polyclonal reference anti-sera, Table S3: Serovar typing was performed by microagglutination tests of isolates with sets of monoclonal antibodies (mAb) corresponding to specific serogroups, Table S4: Summary of cross agglutinin absorption tests (CAAT) performed on strain 782, Table S5: In silico DNA–DNA hybridization of six *L. interrogans* isolates compared with the reference genome *L. interrogans* 43642, Table S6: Assignment of sequence types to the six Malaysian *L. interrogans* strains using MLST (Scheme 1), Table S7: List of *L. interrogans* strains used in SNP genotyping Table S8: Identification and distribution of prophages in six *L. interrogans* strains, Table S9: Identification and distribution of CRISPR spacers in six *L. interrogans* strains, Table S10: Identification and distribution of plasmids in five isolates of *L. interrogans*, Table S11: Identification and distribution of insertion sequence in six *L. interrogans* strains. Table S12: Protein homology of lipid A proteins in six *L. interrogans* strains with reference to strain Fiocruz L1-130, Table S13: Protein homology of sialic acid biosynthesis genes in six *L. interrogans* strains with reference to strain Fiocruz L1-130, Table S14: Protein homologies in the alginate biosynthesis pathway, as well as regulatory and genotypic switching in six *L. interrogans* strains with reference to *P. aeruginosa* PA01, Table S15: Protein Homologies of Bacterial Virulence Factors in Six Strains of *L. interrogans* Blasted in VFDB Software.

## References

[1] Mwachui M.A., Crump L., Hartskeerl R., Zinsstag J., Hattendorf J. (2015). Environmental and behavioural determinants of leptospirosis transmission: A systematic review. PLoS Negl. Trop. Dis..

[2] Andre-Fontaine G., Aviat F., Thorin C. (2015). Waterborne leptospirosis: Survival and preservation of the virulence of pathogenic *Leptospira* spp. in fresh water. Curr. Microbiol..

[3] Costa F., Hagan J.E., Calcagno J., Kane M., Torgerson P., Martinez-Silveira M.S., Stein C., Abela-Ridder B., Ko A.I. (2015). Global morbidity and mortality of leptospirosis: A systematic review. PLoS Negl. Trop. Dis..

[4] Garba B., Bahaman A.R., Khairani-Bejo S., Zakaria Z., Mutalib A.R. (2017). Retrospective study of leptospirosis in Malaysia. EcoHealth.

[5] Vincent A.T., Schiettekatte O., Goarant C., Neela V.K., Bernet E., Thibeaux R., Ismail N., Khalid M.K.N.M., Amran F., Masuzawa T. (2019). Revisiting the taxonomy and evolution of pathogenicity of the genus *Leptospira* through the prism of genomics. PLoS Negl. Trop. Dis..

[6] Cosson J.-F., Picardeau M., Mielcarek M., Tatard C., Chaval Y., Suputtamongkol Y., Buchy P., Jittapalapong S., Herbreteau V., Morand S. (2014). Epidemiology of *Leptospira* transmitted by rodents in Southeast Asia. PLoS Negl. Trop. Dis..

[7] Hochedez P., Theodose R., Olive C., Bourhy P., Hurtrel G., Vignier N., Mehdaoui H., Valentino R., Martinez R., Delord J.-M. (2015). Factors associated with severe leptospirosis, Martinique, 2010–2013. Emerg. Infect. Dis..

[8] Ren S.-X., Fu G., Jiang X.-G., Zeng R., Miao Y.-G., Xu H., Zhang Y.-X., Xiong H., Lu G., Lu L.-F. (2003). Unique physiological and pathogenic features of *Leptospira interrogans* revealed by whole-genome sequencing. Nature.

[9] Picardeau M. (2017). Virulence of the zoonotic agent of leptospirosis: Still terra incognita?. Nat. Rev. Microbiol..

[10] Nascimento A., Ko A.I., Martins E.A.L., Monteiro-Vitorello C.B., Ho P.L., Haake D., Verjovski-Almeida S., Hartskeerl R., Marques M.d.V., Oliveira M.C.d. (2004). Comparative genomics of two *Leptospira interrogans* serovars reveals novel insights into physiology and pathogenesis. J. Bacteriol..

[11] Bulach D.M., Zuerner R.L., Wilson P., Seemann T., McGrath A., Cullen P.A., Davis J., Johnson M., Kuczek E., Alt D.P. (2006). Genome reduction in *Leptospira* borgpetersenii reflects limited transmission potential. Proc. Natl. Acad. Sci. USA.

[12] Zhong Y., Chang X., Cao X.-J., Zhang Y., Zheng H., Zhu Y., Cai C., Cui Z., Zhang Y., Li Y.-Y. (2011). Comparative proteogenomic analysis of the *Leptospira interrogans* virulence-attenuated strain IPAV against the pathogenic strain 56601. Cell Res..

[13] Palaniappan R.U., Chang Y.-F., Jusuf S., Artiushin S., Timoney J.F., McDonough S.P., Barr S.C., Divers T.J., Simpson K.W., McDonough P.L. (2002). Cloning and molecular characterization of an immunogenic LigA protein of *Leptospira interrogans*. Infect. Immun..

[14] Xu Y., Zhu Y., Wang Y., Chang Y.-F., Zhang Y., Jiang X., Zhuang X., Zhu Y., Zhang J., Zeng L. (2016). Whole genome sequencing revealed host adaptation-focused genomic plasticity of pathogenic *Leptospira*. Sci. Rep..

[15] Fouts D.E., Matthias M.A., Adhikarla H., Adler B., Amorim-Santos L., Berg D.E., Bulach D., Buschiazzo A., Chang Y.-F., Galloway R.L. (2016). What makes a bacterial species pathogenic?: Comparative genomic analysis of the genus *Leptospira*. PLoS Negl. Trop. Dis..

[16] Noda A.A., Grillová L., Mariet J.-F., Paiffer N.B., Ruiz Y.C., Rodríguez I., Echevarría E., Obregón A.M., Lienhard R., Picardeau M. (2020). A first insight into the genomic diversity of *Leptospira* strains isolated from patients in Cuba. PLoS ONE.

[17] Santos L.A., Adhikarla H., Yan X., Wang Z., Fouts D.E., Vinetz J.M., Alcantara L.C., Hartskeerl R.A., Goris M.G., Picardeau M. (2018). Genomic Comparison Among Global Isolates of L. interrogans Serovars Copenhageni and Icterohaemorrhagiae Identified Natural Genetic Variation Caused by an Indel. Front. Cell Infect. Microbiol..

[18] Wagenaar J.F., de Vries P.J., Hartskeerl R.A. (2004). Leptospirosis with pulmonary hemorrhage, caused by a new strain of serovar lai: Langkawi. J. Travel Med..

[19] Fukunaga M., Mifuchi I. (1989). Unique organization of *Leptospira interrogans* rRNA genes. J. Bacteriol..

[20] Tindall B. (2014). ATCC 43642 replaces ATCC 23581 as the type strain of *Leptospira interrogans* (Stimson 1907) Wenyon 1926. Opinion 91. Judicial Commission of the International Committee on Systematics of Prokaryotes. Int. J. Syst. Evol. Microbiol..

[21] Boonsilp S., Thaipadungpanit J., Amornchai P., Wuthiekanun V., Bailey M.S., Holden M.T., Zhang C., Jiang X., Koizumi N., Taylor K. (2013). A single multilocus sequence typing (MLST) scheme for seven pathogenic *Leptospira* species. PLoS Negl. Trop. Dis..

[22] Amran F. In Leptospirosis in Malaysia. Proceedings of the 12th Western Pasific Chemotherapy and Infectious Diseases Conference.

[23] Levett P.N. (2015). Systematics of *Leptospira*ceae. Leptospira and Leptospirosis.

[24] Peñafiel E.M., Ramírez F.F., Kawabe L.K. (2015). The use of bacteriophages in the development of new alternatives in therapy. Battle Against Microb. Pathog. Basic Sci. Technol. Adv. Educ. Programs.

[25] Que-Gewirth N.L., Ribeiro A.A., Kalb S.R., Cotter R.J., Bulach D.M., Adler B., Saint Girons I., Werts C., Raetz C.R. (2004). A methylated phosphate group and four amide-linked acyl chains in *Leptospira interrogans* lipid A The membrane anchor of an unusual lipopolysaccharide that activates TLR2. J. Biol. Chem..

[26] Ricaldi J., Matthias M.A., Vinetz J.M., Lewis A.L. (2012). Expression of sialic acids and other nonulosonic acids in *Leptospira*. BMC Microbiol..

[27] Ristow P., Bourhy P., Kerneis S., Schmitt C., Prevost M.-C., Lilenbaum W., Picardeau M. (2008). Biofilm formation by saprophytic and pathogenic leptospires. Microbiology.

[28] Franklin M., Nivens D., Weadge J., Howell P. (2011). Biosynthesis of the Pseudomonas aeruginosa Extracellular Polysaccharides, Alginate, Pel, and Psl. Front. Microbiol..

[29] Murray G.L. (2015). The molecular basis of *Leptospira*l pathogenesis. Leptospira and Leptospirosis.

[30] Bryant J., Chewapreecha C., Bentley S.D. (2012). Developing insights into the mechanisms of evolution of bacterial pathogens from whole-genome sequences. Future Microbiol..

[31] Schiettekatte O., Vincent A.T., Malosse C., Lechat P., Chamot-Rooke J., Veyrier F.J., Picardeau M., Bourhy P. (2018). Characterization of LE3 and LE4, the only lytic phages known to infect the spirochete *Leptospira*. Sci. Rep..

[32] Thakker M., Park J.S., Carey V., Lee J.C. (1998). Staphylococcus aureus serotype 5 capsular polysaccharide is antiphagocytic and enhances bacterial virulence in a murine bacteremia model. Infect. Immun..

[33] Marques M.B., Kasper D.L., Pangburn M.K., Wessels M.R. (1992). Prevention of C3 deposition by capsular polysaccharide is a virulence mechanism of type III group B streptococci. Infect. Immun..

[34] Jerse A.E., Sharma N.D., Simms A.N., Crow E.T., Snyder L.A., Shafer W.M. (2003). A Gonococcal Efflux Pump System Enhances Bacterial Survival in a Female Mouse Model of Genital Tract Infection. Infect. Immun..

[35] Yang F., Yang J., Zhang X., Chen L., Jiang Y., Yan Y., Tang X., Wang J., Xiong Z., Dong J. (2005). Genome dynamics and diversity of Shigella species, the etiologic agents of bacillary dysentery. Nucleic Acids Res..

[36] Chapon-Herve V., Akrim M., Latifi A., Williams P., Lazdunski A., Bally M. (1997). Regulation of the xcp secretion pathway by multiple quorum-sensing modulons in Pseudomonas aeruginosa. Mol. Microbiol..

[37] Stone C.B., Johnson D.L., Bulir D.C., Gilchrist J.D., Mahony J.B. (2008). Characterization of the putative type III secretion ATPase CdsN (Cpn0707) of Chlamydophila pneumoniae. J. Bacteriol..

[38] Ressner R.A., Griffith M.E., Beckius M.L., Pimentel G., Miller R.S., Mende K., Fraser S.L., Galloway R.L., Hospenthal D.R., Murray C.K. (2008). Antimicrobial Susceptibilities of Geographically Diverse Clinical Human Isolates of *Leptospira*. Antimicrob. Agents Chemother..

[39] Siti K.B., Fairuz A., Zunita Z., Aziah D., Mazrura S., Evie K. (2017). Manual for Laboratory Diagnosis of Leptospirosis: One Health Approach.

[40] Zerbino D.R., Birney E. (2008). Velvet: Algorithms for de novo short read assembly using de Bruijn graphs. Genome Res..

[41] Li H., Durbin R. (2009). Fast and accurate short read alignment with Burrows-Wheeler transform. Bioinformatics.

[42] Koboldt D.C., Zhang Q., Larson D.E., Shen D., McLellan M.D., Lin L., Miller C.A., Mardis E.R., Ding L., Wilson R.K. (2012). VarScan 2: Somatic mutation and copy number alteration discovery in cancer by exome sequencing. Genome Res..

[43] Seemann T. (2014). Prokka: Rapid prokaryotic genome annotation. Bioinformatics.

[44] Hartskeerl R. (2006). Leptospirosis: Current status and future trends. Indian J. Med. Microbiol..

[45] (1987). TSCL, Subcomittee on the Taxonomy of *Leptospira*. Int. J. Syst. Bacteriol..

[46] Meier-Kolthoff J.P., Auch A.F., Klenk H.-P., Göker M. (2013). Genome sequence-based species delimitation with confidence intervals and improved distance functions. BMC Bioinform..

[47] Meier-Kolthoff J.P., Göker M. (2019). TYGS is an automated high-throughput platform for state-of-the-art genome-based taxonomy. Nat. Commun..

[48] Treangen T.J., Ondov B.D., Koren S., Phillippy A.M. (2014). The Harvest suite for rapid core-genome alignment and visualization of thousands of intraspecific microbial genomes. Genome Biol..

[49] Kumar S., Stecher G., Tamura K. (2016). MEGA7: Molecular Evolutionary Genetics Analysis Version 7.0 for Bigger Datasets. Mol. Biol. Evol..

[50] Lechner M., Findeiss S., Steiner L., Marz M., Stadler P.F., Prohaska S.J. (2011). Proteinortho: Detection of (co-)orthologs in large-scale analysis. BMC Bioinform..

[51] Bardou P., Mariette J., Escudié F., Djemiel C., Klopp C. (2014). jvenn: An interactive Venn diagram viewer. BMC Bioinform..

[52] Darling A.C., Mau B., Blattner F.R., Perna N.T. (2004). Mauve: Multiple alignment of conserved genomic sequence with rearrangements. Genome Res..

[53] Huerta-Cepas J., Forslund K., Coelho L.P., Szklarczyk D., Jensen L.J., von Mering C., Bork P. (2017). Fast Genome-Wide Functional Annotation through Orthology Assignment by eggNOG-Mapper. Mol. Biol. Evol..

[54] Arndt D., Grant J.R., Marcu A., Sajed T., Pon A., Liang Y., Wishart D.S. (2016). PHASTER: A better, faster version of the PHAST phage search tool. Nucleic Acids Res..

[55] Couvin D., Bernheim A., Toffano-Nioche C., Touchon M., Michalik J., Neron B., Rocha E.P.C., Vergnaud G., Gautheret D., Pourcel C. (2018). CRISPRCasFinder, an update of CRISRFinder, includes a portable version, enhanced performance and integrates search for Cas proteins. Nucleic Acids Res..

[56] Altschul S.F., Madden T.L., Schaffer A.A., Zhang J., Zhang Z., Miller W., Lipman D.J. (1997). Gapped BLAST and PSI-BLAST: A new generation of protein database search programs. Nucleic Acids Res..

[57] Siguier P., Perochon J., Lestrade L., Mahillon J., Chandler M. (2006). ISfinder: The reference centre for bacterial insertion sequences. Nucleic Acids Res..

[58] Zankari E., Hasman H., Cosentino S., Vestergaard M., Rasmussen S., Lund O., Aarestrup F.M., Larsen M.V. (2012). Identification of acquired antimicrobial resistance genes. J. Antimicrob. Chemother..

[59] Sullivan M.J., Petty N.K., Beatson S.A. (2011). Easyfig: A genome comparison visualizer. Bioinformatics.

[60] Chen L., Zheng D., Liu B., Yang J., Jin Q. (2016). VFDB 2016: Hierarchical and refined dataset for big data analysis—10 years on. Nucleic Acids Res..

