# Supplementary material for: Complete Genome Sequencing of Leptospira interrogans Isolates from Malaysia Reveals Massive Genome Rearrangement but High Conservation of Virulence-Associated Genes
Source: Pathogens. 2021 Sep 15;10(9):1198. doi: 10.3390/pathogens10091198 (PMC8467490; doi:10.3390/pathogens10091198)
Supplement: Supplementary file 1 [file pathogens-10-01198-s001.zip › Figure S1_140921_edited.pdf]

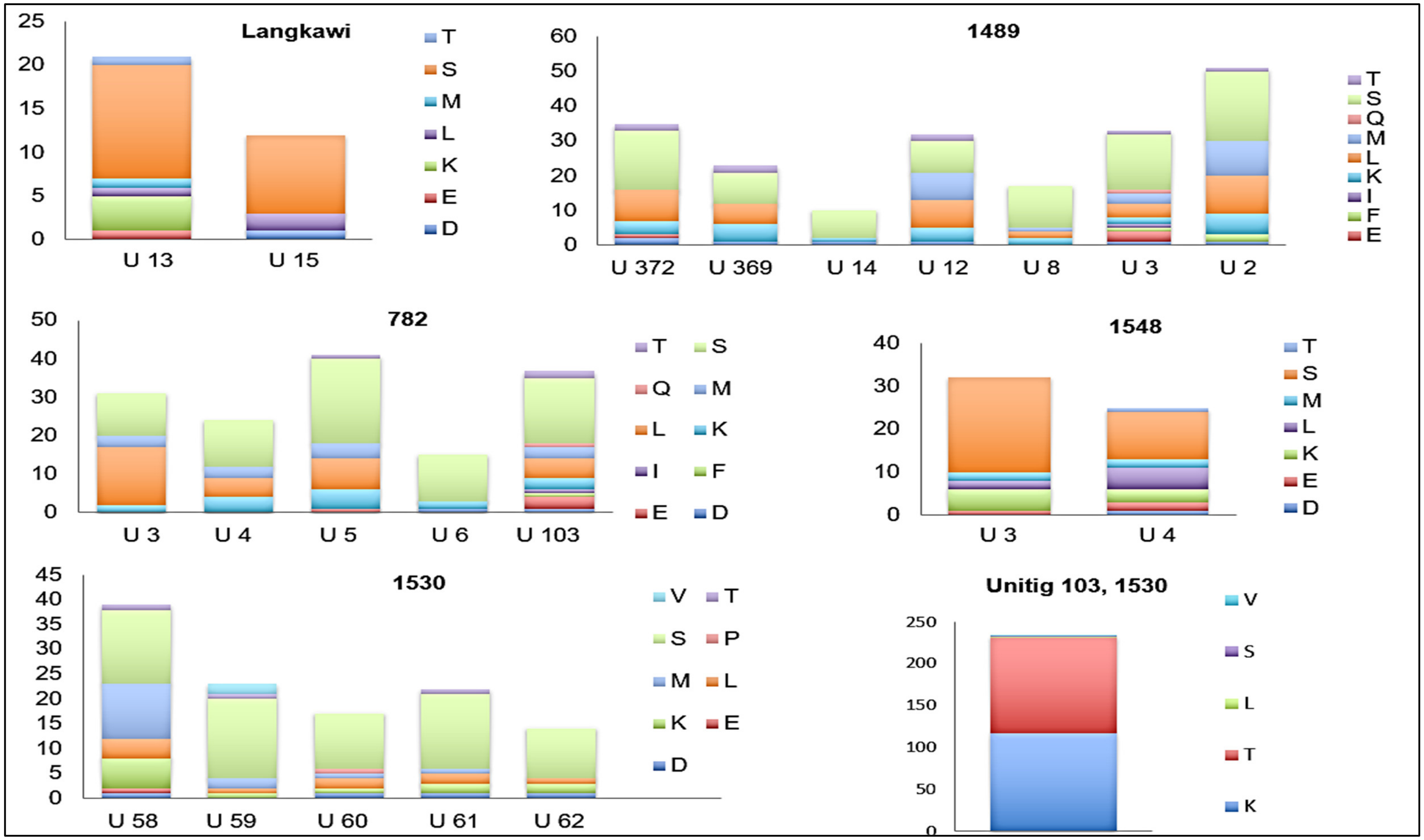

Figure S1. Functional annotation of proteins in the plasmids of the five plasmid-containing *L. interrogans* strains. D: cell cycle control, cell division, chromosome partitioning; E: amino acid transport and metabolism; F: nucleotide transport and metabolism; I: lipid transport and metabolism; K: transcription; L: replication, recombination and repair; M: cell wall, membrane, envelope biogenesis, P: inorganic ion transport and metabolism; Q: secondary metabolites biosynthesis, transport and catabolism; R: general function prediction only; S: function unknown; T: signal transduction mechanism; V: defense mechanism. U = unitig.
