# Supplementary material for: Complete Genome Sequencing of Leptospira interrogans Isolates from Malaysia Reveals Massive Genome Rearrangement but High Conservation of Virulence-Associated Genes
Source: Pathogens. 2021 Sep 15;10(9):1198. doi: 10.3390/pathogens10091198 (PMC8467490; doi:10.3390/pathogens10091198)
Supplement: Supplementary file 1 [file pathogens-10-01198-s001.zip › Figure S2_140921.pdf]

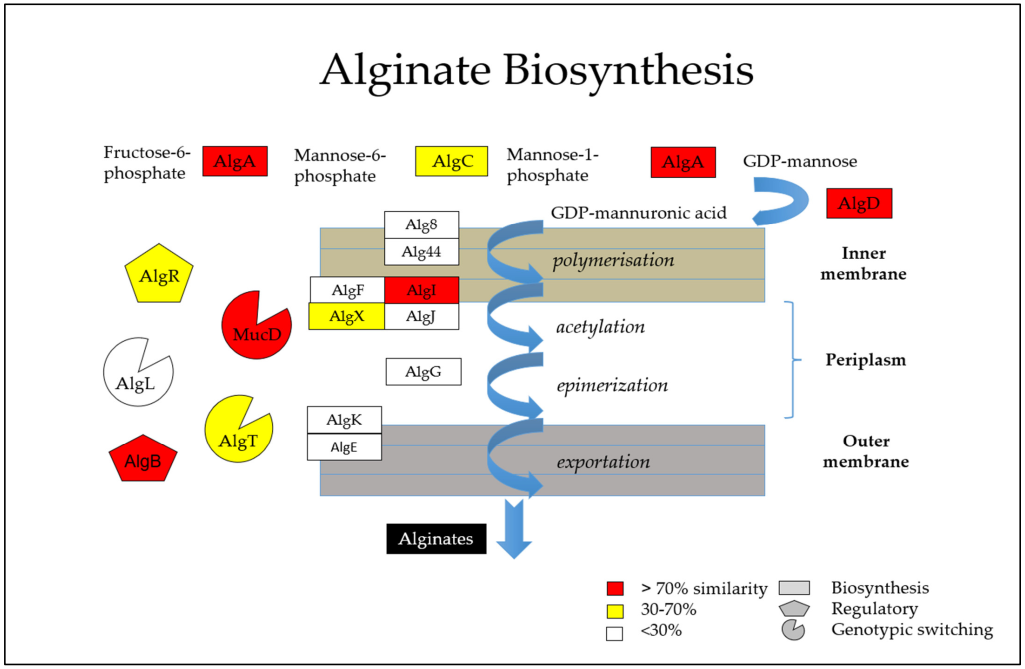

Figure S2. Model of alginate biosynthesis, regulatory and genotypic switching of *L. interrogans* in reference to proteins involved in alginate synthesis in *P. aeruginosa* PA01. Biosynthesis of alginates is initiated by conversion of fructose-6-phosphate to GDP-Mannuronic acid in the cytoplasm catalyzed by the products of *algA*, *algC and algD*. Further conversion, i.e. polymerisation (*alg8* and *alg44*i), acetylation (*algI, algJ, algF and algX*), epimerization (*algG*) and exportation (*algK and algE*), takes places. Protein homology was shown to be high (red) for proteins involved in alginate biosynthesis steps in the cytoplasm (*algA* and *algD*) and inner membranes

(*algI*). There is no sequence homology between *L. interrogans* and *P. aeruginosa* for polymerization (inner membrane), epimerization (periplasmic), and exportation (outer membrane), suggesting different enzymes or mechanisms in *L. interrogans.*
