# Supplementary material for: Complete Genome Sequencing of Leptospira interrogans Isolates from Malaysia Reveals Massive Genome Rearrangement but High Conservation of Virulence-Associated Genes
Source: Pathogens. 2021 Sep 15;10(9):1198. doi: 10.3390/pathogens10091198 (PMC8467490; doi:10.3390/pathogens10091198)
Supplement: Supplementary file 1 [file pathogens-10-01198-s001.zip › Table S1_fp2.pdf]

Table S1. Summary of sociodemographic and clinical data of six patients with *L. interrogans* infection contracted in Malaysia

**Case**	**Langkawi^**	**782**	**898**	**1489**	**1548**	**1530**
Patient details						
Age	50	15	21	24	27	24
Gender	Male	Male	Male	Male	Male	Male
Admission (days)	11	6	6	4	7	6
ICU Admission (days)	2.5	4	0	1	3	0
Clinical details						
History of exposure	Wading mangrove forest 1 week prior to admission	Swimming in river 1 week prior to admission	Rodent infestation at workplace	Swimming in river 2 weeks prior to admission	Patient denied any significant exposure	Jungle trekking and swimming in river prior to admission
Signs symptoms	Fever	High grade fever	Fever	High grade fever	Fever	Fever
	NA	NA	Headache	NA	NA	NA
	NA	chills, rigors	chills, rigors	chills	chills,	chills, rigors,
	myalgia	myalgia, arthralgia	NA	myalgia, arthralgia	myalgia	NA
	nausea, vomiting, diarrhea	epigastric pain	vomiting	vomiting	vomiting epigastric pain, diarrhea	vomiting
	jaundice	jaundice	jaundice	NA	NA	NA
	NA	conjunctival suffusion+	tea-coloured urine	NA	NA	NA

	hemoptysis	dypsnoea	NA	NA	dyspnoea	NA
Complications	Septic shock	Septic shock	NA	Septic shock	Septic shock	NA
	Weil's syndrome,	Weil's syndrome	NA	NA	NA	NA
	pulmonary hemorrhage	NA	NA	NA	NA	NA
	NA	NA	Acute kidney injury	NA	Acute kidney injury	NA
Isolation date & place	Netherlands§ 2004	Klang, Malaysia 2014	Klang, Malaysia 2014	Klang, Malaysia 2014	Klang, Malaysia 2015	Klang, Malaysia 2014
Lab results						
WBC count (>11 x 10^9/L)	16.2	14.9	11.7	11.0	6.5	15
Hemoglobin (<12 g/dL)	ND	11.6	14.6	13	11.2	14
Platelets (<100 x10^9/L)	115	31	127	181	105	184
PT (11 - 13.5 s)	13.7	15.4	ND	ND	ND	ND
INR (0.8 - 1.1)	ND	1.28	ND	ND	ND	ND
CRP (0.8 - 3.0 mg/L)	ND	2161	165	ND	ND	ND
Serum creatinine (>120 µmol/L)	315	124	171	ND	ND	ND
Blood urea (>10 mmol/L)*	ND	9.1	10.1	ND	ND	ND
ALT (>45 U/L)	370	62	22	ND	ND	ND

AST (>40 U/L)	1110	59	80	ND	ND	ND
CK (>190 U/L)	5.421	280	262	ND	ND	ND
Serum bilirubin (17 µmol/L)	ND	29	ND	ND	ND	ND
Bilirubin (mg/dL)	258	ND	65.3	ND	ND	ND
Direct bilirubin (mg/dL	ND	ND	ND	ND	ND	ND
Sodium (135 -145 mEq/L)	ND	136	138	ND	ND	ND
Potassium (3.6 - 5.2 mmol/L)	ND	3.6	3.9	ND	ND	ND
Lepto IgM	1 : 160	Negative	Negative	Negative	Negative	Negative
Dengue IgM	NA	Negative	Negative	Negative	Negative	Negative
Anti-Mycoplasma antibody	NA	NA	NA	NA	1: 40	NA
Vital signs						
SBP (mmHg)	120	88	102	88	130	101
DBP (mmHg)	68	55	62	49	85	60
Pulse rate (min)	124	140	100	79	90	96
Respiratory rate (per min)	ND	35	35	35	35	35
Glasgow Coma Scale	ND	15/15	15/15	15/15	15/15	15/15
Oxygen saturation (SpO_2)	96%	98%	98%	99%	93%	NA
Temperature (ºC)	39	39	38.5	38	39	NA

Treatment						
1st antibiotic	Amoxicillin	Doxycycline	Doxycycline	Doxycycline	Augmentin	Doxycycline
2nd antibiotic	Doxycycline	Ceftriaxone	Ceftriaxone	Ceftriaxone	Azithromycin	Ceftriaxone
Dopamine	Yes	No	NA	No	NA	NA
Inotropes (max)	ND	* Noradr 8 mg	NA	* Noradr 4 mg	NA	NA

All values were determined upon admission to hospital unless stated otherwise. ND, no data; NR, not relevant, ^Wagenaar et al, 2005; ALT, alanine transferase; AST, aspartate trasferase; CK, creatinine kinase; CRP, C-reactive protein; PT, prothrombin time; aPTT, activated partial thromboplastin time; INR, international normalize ratio; WBC, white blood count. *Noradrenaline. §Isolated in the Netherlands, but the infection was contracted in Malaysia.
