# Supplementary material for: Complete Genome Sequencing of Leptospira interrogans Isolates from Malaysia Reveals Massive Genome Rearrangement but High Conservation of Virulence-Associated Genes
Source: Pathogens. 2021 Sep 15;10(9):1198. doi: 10.3390/pathogens10091198 (PMC8467490; doi:10.3390/pathogens10091198)
Supplement: Supplementary file 1 [file pathogens-10-01198-s001.zip › Table S10_fp.pdf]

Table S10. Identification and distribution of plasmids in five isolates of *L. interrogans*.
Same plasmids are identified by same colors. Plasmids were not identified in strain 898.

	Replicon	Size (bp)	Blastn	Score (%)	E-value	Partitioning system
**Langkawi**	Chr 1	437462	*L. interrogans* serovar *Hardjo* str. Norma chromosome 1	99%	0	
	Chr 2	395410	*L. interrogans* serovar *Hardjo* str. Norma chromosome 2	99%	0	
	Plasmid 1	63414	*L. interrogans* serovar *Lai* str. 56601 plasmid Laicp	99%	0	parB
	Plasmid 2	59030	*L. interrogans* serovar *Linhai* str. 56609 plasmid Lcp3	99%	0	parB
**782**	Chr 1	4556406	*L. interrogans* serovar *Lai* str. IPAV chromosome 1	99	0	
	Chr 2	354515	*L. interrogans* serovar *Copenhageni* str. Fiocruz chromosome 2	99	0	
	Plasmid 1	101385	*L. interrogans* serovar *Linhai* str. 56609 chromosome 1	97	0	
	Plasmid 2	90872	*L. interrogans* serovar *Hardjo* str.Norma chromosome 1	97	0	parB
	Plasmid 3	74118	*L. interrogans* serovar *Lai* str. 56601 plasmid *Laicp*	99	0	
	Plasmid 4	53819	*L. interrogans* serovar *Linhai* str. 56609 plasmid Lcp3	91	0	parB
	Plasmid 5	77059	*L. interrogans* serovar *Linhai* str. 56609 plasmid Lcp1	99	0	
**898**	Chr 1	4280412	*L. interrogans* serovar *Copenhageni* str. FDAARGOS chromosome 1	99	0	
	Chr 2	350180	*L. interrogans* serovar *Copenhageni* str. Fiocruz chromosome 2	99	0	
**1489**	Chr 1	4431091	*L. interrogans* serovar *Lai* str. 56601 chromosome 1	99	0	
	Chr 2	353717	*L. interrogans* serovar *Linhai* str. 56609 chromosome 2	99	0	

	Plasmid 1	140106	*L. interrogans* serovar *Hardjo* str.Prajitno chromosome 1	99	0	
	Plasmid 2	83109	*L. interrogans* serovar *Canicola* strain Gui44 plasmid *pGui*1	99	0	
	Plasmid 3	76580	*L. interrogans* serovar *Bratislava* strain PigK151 chromosome 1	99	0	
	Plasmid 4	93306	*L. interrogans* serovar *Linhai* str. 56609 plasmid *lcp*1	99	0	parB
	Plasmid 5	39186	*L .interrogans* serovar *Hardjo* str.Norma chromosome 1	99	0	parB
	Plasmid 6	62980	*L. interrogans* serovar *Linhai* str. 56609 plasmid *lcp*2	99	0	parB
	Plasmid 7	69692	*L. interrogans* serovar *Canicola* strain Gui44 plasmid *pGui*1	99	0	
**1548**	Chr 1	435484	*L. interrogans* serovar *Lai* str. IPAV chromosome 1	99	0	
	Chr 2	393564	*L. interrogans* serovar *Copenhageni* str. FDAARGOS 203 plasmid	99	0	
	Plasmid 1	80435	*L. interrogans* serovar *Manilae* str. UP-MMC-NIID-LP plasmid *pLIMLP*1	99	0	
	Plasmid 2	51265	*L. interrogans* serovar *Linhai* str. 56609 chromosome 1	99	0	parB
**1530**	Chr 1	9684	*L. interrogans* serovar *Copenhageni* str. FDAARGOS chromosome 1	99	0	
	Chr 1	4318326	*L. interrogans* serovar *Hardjo* str.Prajitno chromosome 2	99	0	
	Chr 2	349777	*L. interrogans* serovar *Linhai* str. 56609 chromosome 2	99	0	
	Unitig 58	91738	*L. interrogans* serovar *Linhai* str. 56609 plasmid *lcp*2	99	0	parB
	Unitig 59	82644	*L. interrogans* serovar *Hardjo* str.Norma chromosome 1	99	0	parB
	Unitig 60	52032	*L. interrogans* serovar *Linhai* str. 56609 chromosome 1	99	0	

Unitig 61	54623	*L. interrogans* serovar *Canicola* strain Gui44 plasmid *pGui*1	99	0	parB
Unitig 62	52974	*L. interrogans* serovar *Linhai* str. 56609 plasmid *lcp*3	99	0	parB
Unitig 103	2169	*L. interrogans* serovar *Lai* str. IPAV chromosome 1	99	0	

Chr, chromosome
