# Supplementary material for: Complete Genome Sequencing of Leptospira interrogans Isolates from Malaysia Reveals Massive Genome Rearrangement but High Conservation of Virulence-Associated Genes
Source: Pathogens. 2021 Sep 15;10(9):1198. doi: 10.3390/pathogens10091198 (PMC8467490; doi:10.3390/pathogens10091198)
Supplement: Supplementary file 1 [file pathogens-10-01198-s001.zip › Table S11_fp2.pdf]

Table S11: Identification and distribution of insertion sequence in six *L. interrogans* strains.
Insertion sequence (IS) were identified in the whole genome sequences by ISFinder.

Strain	Replicon	ISLin1	ISLin2	1S500A	IS1500B	IS1501	IS1502	IS1533
**Langkawi**	Chr 1	25	8	ND	ND	ND	1	4
	Chr 2	7	ND	ND	ND	ND	ND	ND
	Plasmid 1	ND	ND	ND	ND	ND	ND	ND
	Plasmid 2	ND	ND	ND	ND	ND	ND	ND
**782**	Chr 1	6	1	ND	ND	ND	ND	ND
	Chr 2	ND	ND	ND	ND	ND	ND	ND
	Plasmid 1	ND	ND	ND	1	ND	ND	ND
	Plasmid 2	ND	ND	ND	ND	1	ND	ND
	Plasmid 3	ND	1	ND	ND	ND	ND	ND
**898**	Chr 1	0	1	8	ND	7	ND	ND
	Chr 2	4	ND	ND	ND	ND	ND	ND
**1489**	Chr 1	91	ND	ND	1	6	2	24
	Chr 2	7	ND	ND	ND	ND	ND	ND
	Plasmid 1	ND	ND	ND	ND	1	ND	ND
	Plasmid 2	ND	3	ND	ND	ND	ND	ND
	Plasmid 3	ND	ND	ND	1	1	ND	ND
	Plasmid 4	ND	ND	1	ND	1	ND	ND
	Plasmid 5	ND	ND	ND	ND	ND	ND	ND
**1548**	Chr 1	65	9	ND	ND	7	2	2
	Chr 2	5	1	ND	ND	ND	ND	ND
	Plasmid 1	ND	ND	ND	ND	1	ND	ND
	Plasmid 2	0	4	ND	ND	ND	ND	ND
**1530**	Chr 1 (unitig_0)	59	8	ND	0	4	1	3
	Chr 2 (unitig_57)	7	ND	ND	1	ND	ND	ND
	Unitig_2	1	ND	ND	ND	1	ND	ND

	Unitig_58	ND	ND	ND	ND	1	ND	ND
	Unitig_59	ND	ND	1	ND	ND	ND	ND
	Unitig_61	ND	1	ND	ND	ND	ND	ND

ND, not detected
Chr, chromosome
