# Supplementary material for: Complete Genome Sequencing of Leptospira interrogans Isolates from Malaysia Reveals Massive Genome Rearrangement but High Conservation of Virulence-Associated Genes
Source: Pathogens. 2021 Sep 15;10(9):1198. doi: 10.3390/pathogens10091198 (PMC8467490; doi:10.3390/pathogens10091198)
Supplement: Supplementary file 1 [file pathogens-10-01198-s001.zip › Table S12_fp2.pdf]

Table S12. Protein homology of lipid A proteins in six *L. interrogans* strains with reference to strain Fiocruz L1-130. The protein homologies of all six *L. interrogans* strains were almost the same except in gene kdsB1, protein LA1625.

Gene locus/ Protein name	GenBank Accession	Protein name	Langkawi	782	898	1489	1548	1530[d]
lpxA / LA_3949	NP_714129.1	UDP-N-acetylglucosamine acyltransferase	100%	100%	100%	100%	100%	100%
lpxC / LA_2306	NP_712487.2	UDP-3-O-[3-hydroxymyristoyl] N-acetylglucosamine deacetylase	99%	99%	99%	100%	100%	100%
lpxD1 / LA_0512	NP_710694.1	UDP-3-O-[3-hydroxymyristoyl] glucosamine N-acyltransferase	100%	100%	100%	100%	100%	100%
lpxD2 / LA_4326	NP_714506.1	UDP-3-O-[3-hydroxymyristoyl]	99%	99%	99%	99%	99%	99%
lpxB1 / LA_0786	NP_710967.1	arabinose kinase	100%	99%	99%	100%	99%	99%
lpxB2 / LA_1096	NP_711277.2	lipid-A-disaccharide synthase	100%	99%	100%	100%	100%	100%
lpxK / LA_3095	NP_713875.1	tetraacyldisaccharide 4'-kinase	100%	99%	99%	99%	99%	100%
kdtA / LA_1477	NP_711657.1	3-deoxy-D-manno-octulosonic-acid transferase	100%	99%	100%	100%	99%	100%
kdsB1 / LA_1625	NP_711806.1	3-deoxy-manno-octulosonate cytidylyltransferase	99%	53%	99%	53%	53%	99%
kdsB2 / LA_3573	NP_713753.2	3-deoxy-manno-octulosonate cytidylyltransferase	100%	100%	100%	100%	100%	100%

lnt / LA_4078	NP_714258.2	apolipoprotein N-acyltransferase	100%	100%	100%	100%	100%	100%
kdsA / LA_2408	NP_712589.2	2-dehydro-3-deoxyphosphooctonate aldolase	100%	100%	100%	100%	100%	100%
htrB / LA_4039	NP_714219.2	lipid A biosynthesis lauroyl acyltransferase	100%	100%	100%	100%	99%	100%
