# Supplementary material for: Complete Genome Sequencing of Leptospira interrogans Isolates from Malaysia Reveals Massive Genome Rearrangement but High Conservation of Virulence-Associated Genes
Source: Pathogens. 2021 Sep 15;10(9):1198. doi: 10.3390/pathogens10091198 (PMC8467490; doi:10.3390/pathogens10091198)
Supplement: Supplementary file 1 [file pathogens-10-01198-s001.zip › Table S13_fp2.pdf]

Table S13. Protein homology of sialic acid biosynthesis genes in six *L. interrogans* strains with reference to strain Fiocruz L1-130. Strains 1489 and 1548 showed differences in 12 out of 13 proteins associated with sialic acids biosynthesis genes.

Protein name	Genebank accession	Function	Langkawi	782	898	1489	1548	1530
LA_1605	NP_711786.1	CMP-N-acetylneuraminic acid synthetase (neuA1)	100%	100%	100%	59%	59%	99%
LA_1606	NP_711787.1	NAD dependent epimerase dehydratase family protein (rfbB3)	100%	100%	100%	93%	93%	99%
LA_1607	NP_711788.1	pyridoxal phosphate dependent aminotransferase	100%	100%	100%	79%	79%	100%
LA_1608	NP_711789.2	sugar acyltransferase, sialic acid O- acetyltransferase NeuD family (neuD)	100%	100%	100%	74%	74%	100%
LA_1609	NP_711790.1	pseudaminic acid synthetase (neuB)	100%	100%	100%	79%	79%	100%
LA_1610	NP_711791.1	UDP-N-acetylglucosamine 2-epimerase (neuC)	100%	100%	100%	78%	78%	100%
LA_1611	NP_711792.1	nucleotidyl transferase	100%	100%	100%	67%	67%	100%
LA_1612	NP_711793.1	hypotheticalprotein	100%	99%	100%	58%	58%	99%
LA_1613	NP_711794.1	N-acetylneuraminic (sialic) acid synthetase (sas/ neuB2)	99%	99%	99%	39%	39%	99%
LA_1614	NP_711795.1	pyridoxal phosphate dependent aminotransferase	100%	100%	100%	52%	52%	100%
LA_1615	NP_711796.1	CMP-N-acetylneuraminic acid synthetase (neuA2)	100%	100%	100%	53%	53%	100%
LA_1645	NP_711826.1	UDP-N- acetylglucosamine 2-epimerase	99%	99%	99%	42%	42%	99%
LA_3823	NP_714003.1	UDP-N-acetylglucosamine diphosphorylase	100%	100%	100%	100%	100%	100%
