# Supplementary material for: Complete Genome Sequencing of Leptospira interrogans Isolates from Malaysia Reveals Massive Genome Rearrangement but High Conservation of Virulence-Associated Genes
Source: Pathogens. 2021 Sep 15;10(9):1198. doi: 10.3390/pathogens10091198 (PMC8467490; doi:10.3390/pathogens10091198)
Supplement: Supplementary file 1 [file pathogens-10-01198-s001.zip › Table S14_fp2.pdf]

Table S14. Protein homologies in the alginate biosynthesis pathway, as well as regulatory and genotypic switching in six *L. interrogans* strains with reference to *P. aeruginosa* PA01. Strong homologies (e-value >56) were detected in 12 proteins associated with structural biosynthesis (including copies of algl), 1 regulatory protein, and 1 protein associated with genotypic switching.

Gene cluster	NCBI	Gene	Gene product	Langkawi	782	898	1489	1548	1530
**Structural/ biosynthetic**	ARG85621.1	*algD*	GDP-mannose dehydrogenase	6e-63	5e-54	3e-63	5e-54	5e-54	5e-54
	OGX66506.1	*alg8*	Subunit of alginate polymerase	ND	ND	ND	ND	ND	ND
	PTC38438.1	*alg44*	Subunit of alginate polymerase	3e (50%)	ND	ND	ND	ND	ND
	AVV63678.1	*algK*	Subunit of protein scaffold	ND	ND	ND	ND	ND	ND
	PTC38436.1	*algE(alg76)*	Porin-like OM protein	ND	ND	ND	ND	ND	ND
	ARG85616.1	*algG*	Mannuronan C-5 epimerase	ND	ND	ND	ND	ND	ND
	AAA91126.1	*algX(alg60)*	Subunit of protein scaffold	3.6 (51%)	4 (51%)	3.4 (51%)	3.4 (51%)	3.6 (51%)	3.6 (51%)
	ARG85614.1	*algL*	Alginate lyase	ND	ND	ND	ND	ND	ND
	ARG85613.1	*algI*	Acetylase	1e-90	1e-90	1e-90	1e-90	1e-90	1e-90
	ARG85613.1	*algI*	Acetylase	2e-83	9e-84	2e-83	2e-83	2e-83	2e-83
	ARG85613.1	*algI*	Acetylase	3e-83	2e-83	3e-83	4e-83	5e-83	3e-83
	ARG85613.1	*algI*	Acetylase	2e-80	2e-80	2e-80	2e-80	2e-80	2e-80
	ARG85613.1	*algI*	Acetylase	5e-80	4e-80	2e-80	4e-80	5e-80	4e-80
	ARG85613.1	*algI*	Acetylase	6e-76	4e-77	4e-77	1e-76	3e-76	4e-76
	ARG85613.1	*algI*	Acetylase	2e-73	1e-73	1e-73	1e-73	9e-74	1e-73

	ARG85613.1	*algI*	Acetylase	4e-73	4e-73	4e-73	4e-73	3e-73	4e-73
	ARG85613.1	*algI*	Acetylase	2e-68	2e-68	3e-64	3e-64	2e-68	3e-64
	ARG85613.1	*algI*	Acetylase	7e-64	8e-64	2e-63	2e-63	9e-64	7e-64
	BAT64151.1	*algJ*	Acetylase	ND	ND	ND	ND	ND	ND
	BAT64150.1	*algF*	Acetylase	ND	ND	ND	ND	ND	ND
	BAR66409.1	*algA*	PMI-GMP	2e-60 (53%)	1e-61 (53%)	3e-62 (53%)	2e-61 (53%)	3e-61 (53%)	3e-61 (53%)
	ARG90425.1	*algC*	PMM	5e-33 (46%)	3e-33 (45%)	3e-33 (45%)	7e-33 (45%)	3e-33 (46%)	3e-33 (46%)
**Regulatory**	AAC44751.1	*algZ(fimS)*	RHH DNA-binding protein	ND	ND	ND	2.0 (60%)	1.8 (60%)	ND
	PTC33675.1	*algR(algR1)*	Response regulator protein of TCSTS	8e-12 (59%)	9e-12 (59%)	6e-12 (59%)	2e-12 (59%)	1.07 (35%)	2e-12 (59%)
	BAR70739.1	*algQ (algR2)*	Cognate sensor kinase	ND	ND	ND	ND	ND	ND
	SIP55675.1	*algP(algR3)*	Histone-like protein	ND	ND	ND	ND	ND	ND
	PTC33420.1	*algB*	NtrC subclass of TCSTS	9e-90 (58%)	1e-89 (58%)	8e-90 (58%)	1e-89 (58%)	9e-90 (58%)	9e-90 (58%)
