# Supplementary material for: Complete Genome Sequencing of Leptospira interrogans Isolates from Malaysia Reveals Massive Genome Rearrangement but High Conservation of Virulence-Associated Genes
Source: Pathogens. 2021 Sep 15;10(9):1198. doi: 10.3390/pathogens10091198 (PMC8467490; doi:10.3390/pathogens10091198)
Supplement: Supplementary file 1 [file pathogens-10-01198-s001.zip › Table S15_fp2.pdf]

Table S15: Protein Homologies of Bacterial Virulence Factors in Six Strains of *L. interrogans* Blasted in VFDB Software

Virulence function	Homologous bacterial virulence factor	Gene	Langkawi	782	898	1489	1548	1530
O	*lipopolysaccharide biosynthesis protein [LPS] [Bordetella pertussis Tohama I]*	*bplC*	9.00E-72	9.00E-72	8.00E-60	4.00E-58	2.00E-71	1.00E-59
O	*lipopolysaccharide biosynthesis protein [LPS] [Bordetella pertussis Tohama I]*	*bplF*	1.00E-94	1.00E-94	1.00E-94	1.00E-94	9.00E-95	1.00E-94
D	*capsular polysaccharide synthesis enzyme Cap8D [Staphylococcus aureus]*	*cap8D*	e-112	e-112	e-112	e-113	e-112	e-122
D	*capsular polysaccharide synthesis enzyme Cap8E [Staphylococcus aureus]*	*cap8E*	e-126	e-126	e-126	9.00E-69	8.00E-69	e-126
D	*capsular polysaccharide synthesis enzyme Cap8F [Staphylococcus aureus]*	*cap8F*	8.00E-92	4.00E-92	8.00E-92	*N/S	*N/S	6.00E-92
D	*capsular polysaccharide synthesis enzyme Cap8G[Staphylococcus aureus]*	*cap8G*	e-115	e-115	e-115	*N/S	*N/S	e-115
N	*cytochrome c heme lyase subunit CcmF [Legionella pneumophila]*	*ccmF*	3.00E-58	3.00E-58	2.00E-55	3.00E-58	9.00E-64	1.00E-63
O	*Type III secretion system ATPase [TTSS] [Chlamydia trachomatis]*	*cdsN*	1.00E-96	1.00E-96	1.00E-96	1.00E-96	1.00E-96	1.00E-96
O	*chemotaxis protein CheA peritrichous flagella [Yersinia enterocolitica]*	*cheA*	3.00E-84	3.00E-83	8.00E-84	2.00E-83	8.00E-84	2.00E-78
O	*chemotaxis histidine kinase [Campylobacter jejuni subsp. jejuni]*	*cheA*	5.00E-93	6.00E-94	6.00E-94	6.00E-94	5.00E-93	5.00E-93
O	*chemotaxis-specific methylesterase CheB [Yersinia enterocolitica]*	*cheB*	1.00E-88	1.00E-88	1.00E-88	1.00E-88	1.00E-88	3.00E-93
O	*chemotaxis-specific methylesterase [Burkholderia pseudomallei]*	*cheB*	2.00E-81	3.00E-69	3.00E-69	3.00E-69	3.00E-69	3.00E-69
D	*endopeptidase Clp ATP-binding chain C [Listeria monocytogenes EGD-e]*	*clpC*	0.0	0.0	0.0	0.0	0.0	0.0
D	*endopeptidase Clp ATP-binding chain C [Listeria monocytogenes EGD-e]*	*clpC*	e-143	0.0	e-142	e-142	e-143	e-143
D	*endopeptidase Clp ATP-binding chain C [Listeria monocytogenes EGD-e]*	*clpC*	0.0	0.0	0.0	0.0	0.0	0.0
D	*ATP-dependent Clp protease proteolytic subunit [Listeria monocytogenes]*	*clpP*	4.00E-57	4.00E-57	4.00E-57	4.00E-57	4.00E-57	4.00E-57
N	*collagenase [kappa-toxin] [Clostridium perfringens str. 13]*	*colA*	2.00E-70	2.00E-70	2.00E-70	2.00E-70	7.00E-71	2.00E-70

N	*glucose-1-phosphate cytidylyltransferase [O-antigen][Yersinia enterocolitica]*	*ddhA*	7.00E-80	7.00E-80	7.00E-80	1.00E-67	1.00E-67	7.00E-80
N	*CDP-glucose 4,6-dehydratase [O-antigen] [Yersinia enterocolitica]*	*ddhB*	8.00E-92	8.00E-92	8.00E-92	4.00E-96	7.00E-96	8.00E-92
N	*GDP-fucose synthetase [O-antigen] [Yersinia enterocolitica]*	*fcl*	e-102	e-102	e-102	7.00E-97	7.00E-97	e-102
N	*ferrous iron transporter B [FeoAB] [Legionella pneumophila]*	*feoB*	2.00E-78	3.00E-78	2.00E-78	3.00E-78	4.00E-78	3.00E-78
O	*flagellar synthesis regulator FleN [polar flagella][Legionella pneumophila]*	*fleN*	3.00E-54	3.00E-54	2.00E-54	3.00E-54	3.00E-54	3.00E-54
O	*transcriptional regulator FleQ [polar flagella] [Legionella pneumophila]*	*fleQ*	2.00E-68	2.00E-68	2.00E-68	2.00E-68	2.00E-68	2.00E-68
O	*sigma 54-dependent response regulator [Legionella pneumophila]*	*fleR/flrC*	5.00E-83	3.00E-83	5.00E-83	5.00E-83	5.00E-83	5.00E-43
O	*flagellar basal-body rod protein [Helicobacter pylori 26695]*	*flgG*	1.00E-74	1.00E-74	1.00E-74	1.00E-74	1.00E-74	1.00E-74
O	*flagellar P-ring protein precursor [Pseudomonas aeruginosa PAO1]*	*flgl*	6.00E-55	8.00E-55	6.00E-55	6.00E-55	6.00E-55	6.00E-55
O	*flagellar biosynthesis protein FlhA [Pseudomonas aeruginosa PAO1]*	*flhA*	e-110	e-110	e-110	e-110	e-110	e-110
O	*flagellar motor switch protein G [Helicobacter pylori]*	*fliG*	3.00E-60	3.00E-60	3.00E-60	3.00E-60	3.00E-60	3.00E-60
O	*flagellar motor switch protein FliM [Helicobacter pylori]*	*fliM*	3.00E-60	3.00E-60	3.00E-60	3.00E-60	3.00E-60	3.00E-60
O	*UDP-glucose 4-epimerase [LOS] [Haemophilus influenzae]*	*galE*	4.00E-58	1.00E-57	1.00E-57	1.00E-57	1.00E-57	1.00E-57
O	*GDP-mannose 4,6-dehydratase [LPS] [Brucella melitensis]*	*gmd*	e-122	e-122	e-122	e-122	e-122	e-122
O	*general secretion pathway protein D [T2SS] [Shigella dysenteriae]*	*gspD*	2.00E-54	2.00E-54	2.00E-54	2.00E-54	2.00E-54	2.00E-54
O	*general secretion pathway protein E [T2SS] [Shigella dysenteriae]*	*gspE*	e-120	e-120	e-120	e-120	e-120	e-120
O	*beta-hemolysin [-hemolysin] [Staphylococcus aureus subsp. aureus]*	*hlb*	2.00E-62	2.00E-57	2.00E-62	2.00E-62	2.00E-57	2.00E-57
O	*beta-hemolysin [-hemolysin] [Staphylococcus aureus subsp. aureus]*	*hlb*	3.00E-55	3.00E-55	3.00E-55	6.00E-52	3.00E-55	3.00E-55
O	*hemolysin transport protein [Hemolysin] [Escherichia coli O157:H7]*	*hlyB*	2.00E-56	2.00E-56	8.00E-56	2.00E-56	2.00E-56	2.00E-56
O	*60K heat shock protein HtpB [Hsp60] [Legionella pneumophila]*	*htpB*	1.00E-169	e-169	e-169	e-169	e-169	e-169
O	*catalase [KatA] [Neisseria meningitidis MC58]*	*katA*	e-157	e-157	e-157	e-157	e-157	e-157
O	*2-dehydro-3-deoxyphosphooctonate aldolase [LPS] [Brucella melitensis]*	*kdsA*	1.00E-71	1.00E-71	1.00E-71	1.00E-71	1.00E-71	1.00E-71
O	*D-arabinose 5-phosphate isomerase [Capsule] [Campylobacter jejuni]*	*kpsF*	*5.00E-61*	5.00E-61	5.00E-61	2.00E-61	1.00E-60	2.00E-61
O	*UDP-N-acetylglucosamine acyltransferase [LOS] [Haemophilus influenzae]*	*lpxA*	2.00E-53	2.00E-53	2.00E-53	2.00E-53	2.00E-53	2.00E-53
O	*lipid-A-disaccharide synthase [LOS] [Haemophilus influenzae Rd KW20]*	*lpxB*	1.00E-53	1.00E-53	2.00E-53	2.00E-53	1.00E-53	2.00E-53

O	*UDP-3-O-(R-3-hydroxymyristoyl)-N-acetylglucosamine deacetylase [LOS] [Haemophilus influenzae]*	*lpxC*	9.00E-53	9.00E-53	9.00E-53	9.00E-53	9.00E-53	9.00E-53
N	*Mg2+ transport protein [MgtBC] [Salmonella enterica]*	*mgtB*	e-133	e-147	e-147	e-147	e-146	e-146
O	*lipid transporter ATP-binding/permease [LOS] [Haemophilus influenzae Rd]*	*msbA*	3.00E-88	2.00E-88	8.00E-88	3.00E-88	3.00E-88	3.00E-88
O	*lipid transporter ATP-binding/permease [LOS] [Haemophilus influenzae Rd]*	*msbA*	5.00E-84	4.00E-84	4.00E-84	4.00E-84	5.00E-84	4.00E-84
D	*multiple transferable resistance system protein MtrD [Neisseria meningitidis MC58]*	*mtrD*	2.00E-56	5.00E-54	2.00E-54	2.00E-56	2.00E-56	2.00E-69
D	*multiple transferable resistance system protein MtrD [Neisseria meningitidis MC58]*	*mtrD*	5.00E-56	2.00E-56	e-56	5.00E-54	5.00E-54	1.00E-91
D	*multiple transferable resistance system protein MtrD [Neisseria meningitidis MC58]*	*mtrD*	2.00E-69	1.00E-69	5.00E-92	2.00E-69	2.00E-69	5.00E-54
D	*multiple transferable resistance system protein MtrD [Neisseria meningitidis MC58]*	*mtrD*	9.00E-92	7.00E-92	5.00E-92	1.00E-91	1.00E-91	2.00E-56
D	*N-acetyl neuramic acid synthetase NeuB [Streptococcus agalactiae]*	*neuB*	5.00E-93	5.00E-93	5.00E-93	7.00E-53	7.00E-53	5.00E-93
D	*N-acetyl neuramic acid synthetase NeuB [Streptococcus agalactiae 2603V/R]*	*neuB*	N	N	N	*2E-91	*2E-91	N
D	*UDP-N-acetylglucosamine-2-epimerase[Streptococcus agalactiae 2603V/R]*	*neuC*	5.00E-65	6.00E-64	5.00E-65	*1E-79	*1E-79	5.00E-65
O	*Perosamine synthetase [LPS] [Brucella melitensis]*	*per*	8.00E-55	8.00E-55	1.00E-54	6.00E-156	4.00E-160	8.00E-55
O	*UDP-N-acetylglucosamine 4,6-dehydratase[Helicobacter pylori 26695]*	*pseB*	8.00E-66	8.00E-66	7.00E-66	*2E-97	*2E-97	8.00E-66
O	*C4 aminotransferase [Campylobacter jejuni subsp. jejuni NCTC 11168]*	*pseC*	N	N	N	*4E-65	*4E-65	N
O	*N-acetylneuraminic acid synthetase[Campylobacter jejuni.]*	*psel*	8.00E-52	6.00E-52	8.00E-52	*8E-65	*7E-65	8.00E-52
D	*DNA repair protein RecN [RecN] [Neisseria meningitidis]*	*recN*	7.00E-66	7.00E-66	7.00E-66	7.00E-66	7.00E-66	7.00E-66
R	*probable GTP pyrophosphokinase RelA [Mycobacterium tuberculosis]*	*relA*	e-149	e-149	e-149	e-149	e-149	e-149
O	*ADP-L-glycero-D-mannoheptose-6-epimerase [Haemophilus influenzae]*	*rfaD*	2.00E-57	4.00E-58	4.00E-58	4.00E-58	3.00E-57	1.00E-57
O	*dTDP-glucose 46-dehydratase [LOS] [Haemophilus influenzae]*	*rffG*	2.00E-97	2.00E-97	2.00E-97	2.00E-97	1.00E-97	2.00E-97
N	*sphingomyelinase-c [SMase] [Listeria ivanovii]*	*smcL*	1.00E-59	1.00E-59	2.00E-59	1.00E-59	1.00E-59	1.00E-59
N	*sphingomyelinase-c [SMase] [Listeria ivanovii]*	*smcL*	1.00E-69	1.00E-69	4.00E-69	1.00E-69	1.00E-69	1.00E-69

O	*general secretion pathway protein F [Pseudomonas aeruginosa]*	*xcpS*	2.00E-56	2.00E-56	2.00E-56	2.00E-56	2.00E-56	2.00E-56
O	*phosphomannomutase [LOS] [Haemophilus influenzae]*	*yhxB/manB*	1.00E-62	6.00E-63	6.00E-63	2.00E-62	6.00E-63	2.00E-65
O	beta-hemolysin [-hemolysin] [*Staphylococcus aureus*]	*hlb*	2.00E-62	2.00E-57	2.00E-62	2.00E-62	2.00E-57	2.00E-57
O	beta-hemolysin [-hemolysin] [*Staphylococcus aureus* subsp. aureus COL]	*hlb*	3.00E-55	3.00E-55	3.00E-55	6.00E-52	3.00E-55	3.00E-55
O	hemolysin transport protein [Hemolysin] [*Escherichia coli* O157:H7 str. EDL933]	*hlyB*	2.00E-56	2.00E-56	8.00E-56	2.00E-56	2.00E-56	2.00E-56
O	Hsp60, 60K heat shock protein HtpB [Hsp60] [*Legionella pneumophila*]	*htpB*	1.00E-169	e-169	e-169	e-169	e-169	e-169
O	catalase [KatA] [*Neisseria meningitidis* MC58]	*katA*	e-157	e-157	e-157	e-157	e-157	e-157
O	2-dehydro-3-deoxyphosphooctonate aldolase [LPS] [*Brucella melitensis*]	*kdsA*	1.00E-71	1.00E-71	1.00E-71	1.00E-71	1.00E-71	1.00E-71
O	D-arabinose 5-phosphate isomerase [*Campylobacter jejuni*]	*kpsF*	*5.00E-61*	5.00E-61	5.00E-61	2.00E-61	1.00E-60	2.00E-61
O	UDP-N-acetylglucosamine acyltransferase [*Haemophilus influenzae*]	*lpxA*	2.00E-53	2.00E-53	2.00E-53	2.00E-53	2.00E-53	2.00E-53
O	lipid-A-disaccharide synthase [LOS] [*Haemophilus influenzae*]	*lpxB*	1.00E-53	1.00E-53	2.00E-53	2.00E-53	1.00E-53	2.00E-53
O	UDP-3-O-N-acetylglucosamine deacetylase [*Haemophilus influenzae*]	*lpxC*	9.00E-53	9.00E-53	9.00E-53	9.00E-53	9.00E-53	9.00E-53
N	Mg2+ transport protein [MgtBC] [*Salmonella enterica*]	*mgtB*	e-133	e-147	e-147	e-147	e-146	e-146
O	lipid transporter ATP-binding/permease [LOS] [*Haemophilus influenzae*]	*msbA*	3.00E-88	2.00E-88	8.00E-88	3.00E-88	3.00E-88	3.00E-88
O	lipid transporter ATP-binding/permease [LOS] [*Haemophilus influenzae*]	*msbA*	5.00E-84	4.00E-84	4.00E-84	4.00E-84	5.00E-84	4.00E-84
D	multiple transferable resistance system protein MtrD [*Neisseria meningitidis*]	*mtrD*	2.00E-56	5.00E-54	2.00E-54	2.00E-56	2.00E-56	2.00E-69
D	multiple transferable resistance system protein MtrD [*Neisseria meningitidis*]	*mtrD*	5.00E-56	2.00E-56	e-56	5.00E-54	5.00E-54	1.00E-91
D	multiple transferable resistance system protein MtrD [*Neisseria meningitidis*]	*mtrD*	2.00E-69	1.00E-69	5.00E-92	2.00E-69	2.00E-69	5.00E-54
D	multiple transferable resistance system protein MtrD [*Neisseria meningitidis*]	*mtrD*	9.00E-92	7.00E-92	5.00E-92	1.00E-91	1.00E-91	2.00E-56
D	N-acetyl neuramic acid synthetase NeuB [Capsule][*Streptococcus agalactiae*]	*neuB*	5.00E-93	5.00E-93	5.00E-93	7.00E-53	7.00E-53	5.00E-93

D	N-acetyl neuramic acid synthetase NeuB [Capsule]*[Streptococcus agalactiae*]	*neuB*	N	N	N	*2E-91	*2E-91	N
D	UDP-N-acetylglucosamine-2-epimerase NeuC [*Streptococcus agalactiae* 2603V/R]	*neuC*	5.00E-65	6.00E-64	5.00E-65	*1E-79	*1E-79	5.00E-65
O	Perosamine synthetase [LPS] [*Brucella melitensis*]	*per*	8.00E-55	8.00E-55	1.00E-54	6.00E-156	4.00E-160	8.00E-55
O	UDP-N-acetylglucosamine 4,6-dehydratase [*Helicobacter pylori*]	*pseB*	8.00E-66	8.00E-66	7.00E-66	*2E-97	*2E-97	8.00E-66
O	C4 aminotransferase [*Campylobacter jejuni]*	*pseC*	N	N	N	*4E-65	*4E-65	N
O	N-acetylneuraminic acid synthetase [*Campylobacter jejuni*]	*psel*	8.00E-52	6.00E-52	8.00E-52	*8E-65	*7E-65	8.00E-52
D	DNA repair protein RecN [RecN] [*Neisseria meningitidis*]	*recN*	7.00E-66	7.00E-66	7.00E-66	7.00E-66	7.00E-66	7.00E-66
R	probable GTP pyrophosphokinase [*Mycobacterium tuberculosis*]	*relA*	e-149	e-149	e-149	e-149	e-149	e-149
O	*ADP-L-glycero-D-mannoheptose-6-epimerase [Haemophilus influenzae*]	*rfaD*	2.00E-57	4.00E-58	4.00E-58	4.00E-58	3.00E-57	1.00E-57
O	dTDP-glucose 46-dehydratase [LOS] [*Haemophilus influenzae*]	*rffG*	2.00E-97	2.00E-97	2.00E-97	2.00E-97	1.00E-97	2.00E-97
N	sphingomyelinase-c [SMase] [*Listeria ivanovii*]	*smcL*	1.00E-59	1.00E-59	2.00E-59	1.00E-59	1.00E-59	1.00E-59
N	sphingomyelinase-c [SMase] [*Listeria ivanovii*]	*smcL*	1.00E-69	1.00E-69	4.00E-69	1.00E-69	1.00E-69	1.00E-69
O	general secretion pathway protein F [*Pseudomonas aeruginosa*]	*xcpS*	2.00E-56	2.00E-56	2.00E-56	2.00E-56	2.00E-56	2.00E-56
O	phosphomannomutase [LOS] [*Haemophilus influenzae*]	*yhxB/manB*	1.00E-62	6.00E-63	6.00E-63	2.00E-62	6.00E-63	2.00E-65

N, blast result ≥ e-value 1e-52 or score of alignment ≤ 200 (ie. not significant)

*Similarities found only between 1489 and 1548

Green (D) defensive virulence factors; red (O) offensive virulence factors; pink (R) regulatory; yellow (N) nonspecific.
