# Supplementary material for: Complete Genome Sequencing of Leptospira interrogans Isolates from Malaysia Reveals Massive Genome Rearrangement but High Conservation of Virulence-Associated Genes
Source: Pathogens. 2021 Sep 15;10(9):1198. doi: 10.3390/pathogens10091198 (PMC8467490; doi:10.3390/pathogens10091198)
Supplement: Supplementary file 1 [file pathogens-10-01198-s001.zip › Table S2_fp2.pdf]

Table S2: Summary of serogroup typing by microscopic agglutination test with polyclonal reference anti-sera§

Strain	Serogroup typing with reference polyclonal anti-sera	Titre
**782**	Serogroup Ballum, serovar *Kenya*, strain Njenga	1:80
	Serogroup Canicola, serovar *Canicola,* strain Hond Utrecht IV	1:1280
	Serogroup Icterohaemorrhagiae, serovar *Copenhageni,* strain Wijnberg	1:40
	Serogroup Javanica, serovar *Poi*, strain Poi	1:80
**898**	Serogroup Icterohaemorrhagiae, serovar *Copenhageni,* strain M20	1:10240
	Serogroup Icterohaemorrhagiae, serovar *Icterohaemorrhagiae,* strain RGA	1:10240
	Serogroup Sarmin, serovar *Weaveri*, strain CZ 390	1:1280
**1489**	Serogroup Bataviae, serovar *Bataviae*, strain Swart	1:2560
	Serogroup Djasiman, serovar *Djasiman*, strain Djasiman	1:80
**1548**	Serogroup Bataviae, serovar *Bataviae*, strain Swart	1:2560
	Serogroup Tarassovi, serovar *Rama,* strain 316	1:1520
	Serogroup Tarassovi, serovar *Tarassovi,* strain Perepelitsin	1:160
**1530**	Serogroup Icterohaemorrhagiae, serovar *Copenhageni,* strain M20	1:10240
	Serogroup Icterohaemorrhagiae, serovar *Icterohaemorrhagiae,* strain RGA	1:320
	Serogroup Pyrogenes, serovar *Pyrogenes*, strain Salinem	1:80
	Serogroup Sarmin, serovar *Weaveri*, strain CZ 390	1:640

§ Australis, Autumnalis, Ballum, Bataviae, Canicola, Celledoni, Cynopteri, Djasiman, Grippotyphosa, Hebdomadis, Icterohaemorrhagiae Javanica, Louisiana, Manhao, Mini, Panama, Pomona, Pyrogenes, Sarmin, Sejroe, Shermani, Tarassovi, Semaranga, Hurstbridge, Leptonema, Ranarum,Genomospecies 1 Sichuan 79601, Difco contaminant, Lyme.
