# Supplementary material for: Complete Genome Sequencing of Leptospira interrogans Isolates from Malaysia Reveals Massive Genome Rearrangement but High Conservation of Virulence-Associated Genes
Source: Pathogens. 2021 Sep 15;10(9):1198. doi: 10.3390/pathogens10091198 (PMC8467490; doi:10.3390/pathogens10091198)
Supplement: Supplementary file 1 [file pathogens-10-01198-s001.zip › Table S3_SR_fp (1).pdf]

Table S3. Serovar typing was performed by microagglutination tests of isolates with sets of monoclonal antibodies (mAb) corresponding to specific serogroups. Five strains could be assigned to serovars but results for strain 782 were inconclusive.

**Strain**	**782**	**898**	**1489**	**1548**	**1530**
**Serogroup**	Canicola	Icterohaemorrhagiae	Bataviae	Bataviae	Icterohaemorrhagiae
**mAb panel**	F152C1; 80<br>F152C2; 10<br>F152C5; 10<br>F152C7; 5120<br>F152C8; 640<br>F152C10; 20480<br>F152C11; 2560<br>F152C13; 10<br>F152C14; 2560<br>F152C17; 10<br>F152C18; 10	F20C3-1; 10240<br>F20C4-3; 20480<br>F52C1-4; 80<br>F52C2-4; 10<br>F70C4-5; 2560<br>F70C13-5; 1280<br>F70C20-4; 2560<br>F70C24-20; 2560<br>F70C26-3; 1280<br>F82C1-3; 10<br>F82C2-3; 10<br>F82C7-3; 10<br>F82C8-4; 10<br>F89C3-3; 320<br>F89C12-6; 640	F129C2-3; 20480<br>F129C3-3; 5120<br>F129C4-1; 640<br>F129C6-1; 1280<br>F129C7-1; 320<br>F129C9-5; 10<br>F129C15-3; 10<br>F129C18-1; 10<br>F129C19-3; 20480<br>F129C20-5; 20480<br>F129C24-3; 20480<br>F129C25-1; 2560<br>F129C26-1; 10	F129C2-3; 20480<br>F129C3-3; 10240<br>F129C4-1; 320<br>F129C6-1; 20480<br>F129C7-1; 20480<br>F129C9-5; 5120<br>F129C15-3; 5120<br>F129C18-1; 20480<br>F129C19-3; 20480<br>F129C20-5; 20480<br>F129C24-3; 20480<br>F129C25-1; 20480<br>F129C26-1; 1280	F20C3-1; 10<br>F20C4-3; 10<br>F52C1-4; 160<br>F52C2-4; 10<br>F70C4-5; 1280<br>F70C13-5; 2560<br>F70C20-4; 640<br>F70C24-20; 10<br>F70C26-3; 1280<br>F82C1-3; 1280<br>F82C2-3; 40<br>F82C7-3; 10<br>F82C8-7; 5120<br>F89C3-3; 5120<br>F89C12-6; 5120
**Homologous serovar**	To be determined by CAAT	serovar Copenhageni / Icterohaemorrhagiae	serovar Paidjan, strain Paidjan	serovar Bataviae, strain Swart	serovar Yeonchon, strain HM 3
