# Supplementary material for: Complete Genome Sequencing of Leptospira interrogans Isolates from Malaysia Reveals Massive Genome Rearrangement but High Conservation of Virulence-Associated Genes
Source: Pathogens. 2021 Sep 15;10(9):1198. doi: 10.3390/pathogens10091198 (PMC8467490; doi:10.3390/pathogens10091198)
Supplement: Supplementary file 1 [file pathogens-10-01198-s001.zip › Table S4_fp2.pdf]

Table S4. Summary of cross agglutinin absorption tests (CAAT) performed on strain 782.

Antiserum	Antigen	Cross-agglutination (%)	CAAT, live antigen (%)	CAAT, dead antigen (%)
**Bafani**	782	6.25	Nil	Nil
**Benjamini**	782	3.13	Nil	Nil
**Malaya H6**	782	< 3.13	Nil	Nil
**Galtoni**	782	6.25	Nil	Nil
**Kuwait**	782	3.13	Nil	Nil
**Vleermus qoc**	782	6.25	Nil	Nil
**Qunjian**	782	3.13	Nil	Nil
**Hond Utrecht**	782	50	25	25
**Yonsis**	782	50	25	25
**Portlandvere**	782	50	25	50
**Sumneri**	782	50	25	25
**Kamituga**	782	12/62*	Nil	Nil
**782**	Kamituga	12/62*	100	50
**Broomi patane**	782	50	0.2	0.2
**782**	Broomi patane	50	6.3	6.3
**782**	Broomi patane	50	12.5	12.5
**Broomi patane**	782	50	3.1	3.1
**782**	Broomi patane	50	12.5	12.5
**782**	Broomi patane	50	12.5	12.5
**Bindjei**	782	50	0.8	< 0.4
**782**	Bindjei	50	6.3	0.4
**782**	Bindjei	50	3.1	6.3
**Bindjei**	782	50	3.1	1.6
**782**	Bindjei	50	1.6	1.6

*Results were inconclusive, as repeat measures yielded both values.
