# Supplementary material for: Complete Genome Sequencing of Leptospira interrogans Isolates from Malaysia Reveals Massive Genome Rearrangement but High Conservation of Virulence-Associated Genes
Source: Pathogens. 2021 Sep 15;10(9):1198. doi: 10.3390/pathogens10091198 (PMC8467490; doi:10.3390/pathogens10091198)
Supplement: Supplementary file 1 [file pathogens-10-01198-s001.zip › Table S5_fp2.pdf]

Table S5. ***In silico*** **DNA-DNA hybridization (DDH) of six** ***L. interrogans*** **isolates compared with the reference genome** ***L. interrogans*** **ATCC 43642.**

Reference genome	DDH	Model confidence interval	Distance	Probable DDH ≥ 70%	G+C difference
**Langkawi**	93	[91.1 - 94.6%]	0.0088	96.71	0.21
**782**	92.8	[90.8 - 94.3%]	0.0091	96.64	0.19
**898**	98.4	[97.7 - 98.9%]	0.0025	97.95	0.03
**1489**	92.2	[90.2 - 93.9%]	0.0097	96.49	0.13
**1548**	91.3	[89.2 - 93.1%]	0.0107	96.22	0.15
**1530**	93.4	[91.5 - 94.9%]	0.0084	96.8	0.01
