# Supplementary material for: Complete Genome Sequencing of Leptospira interrogans Isolates from Malaysia Reveals Massive Genome Rearrangement but High Conservation of Virulence-Associated Genes
Source: Pathogens. 2021 Sep 15;10(9):1198. doi: 10.3390/pathogens10091198 (PMC8467490; doi:10.3390/pathogens10091198)
Supplement: Supplementary file 1 [file pathogens-10-01198-s001.zip › Table S6_fp2.pdf]

Table S6. Assignment of sequence types to the six Malaysian *L. interrogans* strains using MLST (Scheme 1).
Sequence types were assigned using the *Leptospira* MLST website (http://leptospira.mlst. net/).

Isolate	*glmU*	*pntA*	*sucA*	*tpiA*	*pfkB*	*mreA*	*caiB*	ST	Serovar/strain
**Langkawi**	1	71^	1	13	34	3	6	236*	NR
**782**	1	13	2	2	6	10	13	240*	NR
**898**	1	1	2	2	10	4	8	17	*Copenhageni* Fiocruz L1-130/ *Icterohaemorrhagiae* RGA
**1489**	1	1	1	4	18	5	6	241*	NR
**1548**	6	8	2	2	9	7	5	50	*Bataviae* / Losbanos LT101-69
**1530**	1	1	1	1	76^	2	58^	237*	NR

^ New allele

* Novel ST

NR, not relevant
