# Supplementary material for: Complete Genome Sequencing of Leptospira interrogans Isolates from Malaysia Reveals Massive Genome Rearrangement but High Conservation of Virulence-Associated Genes
Source: Pathogens. 2021 Sep 15;10(9):1198. doi: 10.3390/pathogens10091198 (PMC8467490; doi:10.3390/pathogens10091198)
Supplement: Supplementary file 1 [file pathogens-10-01198-s001.zip › Table S7_fp2.pdf]

Table S7. List of *L. interrogans* strains used in SNP genotyping. Whole genome sequences of eight strains were downloaded from NCBI GeneBank. 15 other Malaysian strains were obtained from Leptospira Reference Center, Amsterdam, and draft genomes were generated in this study.

No	Serovar	Strain	Source
1	Lai	56601	NCBI
2	Lai	IPAV	NCBI
3	Hardjo	Norma	NCBI
4	Manilae	NIID-HP	NCBI
5	Manilae	NIID-LP	NCBI
6	Copenhageni	Fiocruz L1-130	NCBI
7	Linhai	56609	NCBI
8	Bratislave	PigK151	NCBI
9	Abramis	Abraham	This study
10	Autumnalis	Akiyami A	This study
11	Australis	Ballico	This study
12	Bataviae	Swart	This study
13	Biggis	Biggs	This study
14	Birkini	Birkin	This study
15	Djasiman	Djasiman	This study
16	Evansi	267-1348	This study
17	Gurungi	Gurung	This study
18	Haemolytica	Sejroe	This study
19	Icterohaemorhagiae	RGA	This study
20	Muelleri	RM2	This study
21	Pomona	Pomona	This study
22	Ricardi	Richarson	This study
23	Smithi	Smith	This study
