# Supplementary material for: Complete Genome Sequencing of Leptospira interrogans Isolates from Malaysia Reveals Massive Genome Rearrangement but High Conservation of Virulence-Associated Genes
Source: Pathogens. 2021 Sep 15;10(9):1198. doi: 10.3390/pathogens10091198 (PMC8467490; doi:10.3390/pathogens10091198)
Supplement: Supplementary file 1 [file pathogens-10-01198-s001.zip › Table S8_fp2.pdf]

Table S8. Identification and distribution of prophages in six *L. interrogans* strains.

Strain	Replicon	Region length	Completeness	Score	No. Total Proteins	Region position	Phage identification	Detail
**Langkawi**	Chr 2	8.6Kb	incomplete	30	12	237357-246004	PHAGE_Salisa_1_	terminase, tail
	Chr 1	9.7Kb	incomplete	60	11	1047304-1057067	PHAGE_Lister_B054_	head, capsid
	Chr 1	10.5Kb	incomplete	30	14	1058927-1069497	PHAGE_Deep_s_D6E_	plate, tail
	Chr 1	9.4Kb	incomplete	10	10	1426160-1435647	PHAGE_Bacill_SP_15_	-
	Chr 1	11.4Kb	incomplete	10	9	2621637-2633036	PHAGE_Sphing_PAU_	-
	Chr 1	6.9Kb	incomplete	30	7	2709383-2716331	PHAGE_Synech_S_CAM7_	lysis
	Chr 1	6.2Kb	incomplete	40	8	3211720-3217923	PHAGE_Pseudo_LMA2_	tail, plate
**782**	Chr 1	23Kb	incomplete	30	13	1155136-1178139	PHAGE_Bacill_PfEFR_5_	transposase
	Chr 1	7.2Kb	incomplete	50	9	1226026-1233243	PHAGE_Pseudo_LMA2_	plate, tail
	Chr 1	5Kb	incomplete	50	8	1628022-1633023	PHAGE_Bacill_SP_15_	transposase
	Chr 1	8.6Kb	incomplete	30	11	2641470-2650117	PHAGE_Salisa_1_	terminase,tail

	Chr 1	11.3Kb	incomplete	10	9	2772751-2784143	PHAGE_Sphing_PAU	-
	Chr 1	6.9Kb	incomplete	30	7	2867731-2874679	PHAGE_Synech_S_CAM7_	lysis
	Chr 1	9.4Kb	incomplete	10	10	3077867-3087353	PHAGE_Synech_ACG_2014b_	-
	Chr 1	9.9Kb	incomplete	30	13	3161600-3171587	PHAGE_Psychr_pOW20_A	tail
	Chr 1	8.6Kb	incomplete	30	12	3337482-3346129	PHAGE_Salisa_1_	terminase,tail
	Plasmid 3	5.8Kb	incomplete	20	11	30880-36691	PHAGE_Mannhe_vB_MhS_535AP2	tail
**1489**	Chr 1	28.3Kb	incomplete	60	33	1730220-1758602	PHAGE_Sphing_PAU_	transposase
**898**	Chr 1	9.4Kb	incomplete	10	10	1386689-1396161	PHAGE_Bacill_SP_15_	-
	Chr 1	11.4Kb	incomplete	10	10	2563962-2575364	PHAGE_Sphing_PAU_	-
	Chr 1	11.6Kb	incomplete	30	11	2647794-2659399	PHAGE_Synech_S_CAM7_	lysis
	Chr 1	6.2Kb	incomplete	40	8	3153143-3159346	PHAGE_Pseudo_LMA2_	tail, plate
**1548**	Chr 1	8.4Kb	incomplete	30	9	3037136-3045545	PHAGE_Acidia_virus_	transposase
**1530**	Chr 1	6.2Kb	incomplete	40	8	308140-314343	PHAGE_Pseudo_LMA2_	-
	Chr 1	7Kb	incomplete	30	7	2538121-2545213	PHAGE_Lactob_Lj965_	tail, plate

	Chr 1	9.4Kb	incomplete	10	11	2813059-2822530	PHAGE_Bacill_SP_15_	transposase
	Chr 1	11.3Kb	incomplete	10	9	4024631-4036023	PHAGE_Sphing_PAU_	-
	Chr 1	11.6Kb	incomplete	30	11	4109976-4121581	PHAGE_Synech_S_CAM7_	lysis

Chr, chromosome
