# Supplementary material for: Complete Genome Sequencing of Leptospira interrogans Isolates from Malaysia Reveals Massive Genome Rearrangement but High Conservation of Virulence-Associated Genes
Source: Pathogens. 2021 Sep 15;10(9):1198. doi: 10.3390/pathogens10091198 (PMC8467490; doi:10.3390/pathogens10091198)
Supplement: Supplementary file 1 [file pathogens-10-01198-s001.zip › Table S9_fp2SR2_fp (1).pdf]

Table S9. Identification and distribution of CRISPR spacers in six *L. interrogans* strains

Strain	Repeat Region	Copy	CRSIPR	Blastn (spacer)	Coverage (%)	Identification (%)	Plasmid match
**Langkawi**	DR consensus	1	CGCCTAACGGCATCAAAGTT ATATTCAG	*Leptospira interrogans* serovar Linhai str.56609"plasmid *lcp3*	81	100	None
	Spacer	2	AAAATCGTTGTTGCGAAATG CGGATCGTTGTTCAGGTGCT CAA	product = "putative bacteriophage tail fiber protein"			
	DR consensus	4	CGCCTAACGGCATCAAAGTT ATATTCAG	*Leptospira interrogans* strain FMASAW1 plasmid *pLiSL*2	81	100	None
	Spacer	1	CTTGAATCGAAGTGTTAGAA ATTGAAAATTTTTTCGGTGCT CAA	product="hypothetical protein"			
	DR consensus	6	CGCCTAACGGCATCAAAGTT ATATTCAG	*Leptospira interrogans* serovar Linhai str.56609"plasmid *lcp3*,	80	100	None
**Total CRISPR:16**	Spacer	5	TTCGTCAAAGACGAGAGAG TATCTCAAGAGAAGAGTGCT CAA	product="Helix-turn-helix domain containing protein"			
**782**	DR consensus	12	CTGAATATAACTTTGATGCC GTTAGGCGTTGAGCAC	*Leptospira interrogans* serovar Linhai	100	97.14	None

				str.56609"plasmid *lcp1,*			
	Spacer	1	ATCATGCGAATCCGGTGGG AACGTTGCTGGTTTAT	product="putative bacteriophage tail fiber protein"			
	DR consensus	12	CTGAATATAACTTTGATG CCGTTAGGCGTTGAGCAC	*Leptospira interrogans* serovar Linhai str.56609" plasmid *lcp3,*	100	100	None
	Spacer	2	ATCCAAATTGTCCTTTCCCC ATTGCCTGTAGGGAT	product="hypothetical protein"			
	DR consensus	12	CTGAATATAACTTTGATGCC GTTAGGCGTTGAGCAC	*Leptospira interrogans* serovar Linhai str.56609"plasmid *lcp3*,	100	100	Langkawi: Unitig 15; 1530: Unitig 62; 782: Unitig 6.
	Spacer	3	CAATTGTGATCGGAATCGGT TTCCAAAATTTAAG	product="putative phage replication related protein"			
	DR consensus	12	CTGAATATAACTTTGATGCC GTTAGGCGTTGAGCAC	*Leptospira interrogans* strain FMAS_AW1 plasmid p*LiSL2*	100	100	None
	Spacer	5	GATGAAACAAAAGTCCCTC GAATTCAAGTGAGGCT	product="hypothetical protein"			
	DR consensus	12	CTGAATATAACTTTGATGCC GTTAGGCGTTGAGCAC	*Leptospira interrogans* strain FMAS_AW1 plasmid *pLiSL2*	100	100	None
	Spacer	7	AGGTCCACCCCTTCCTGAGT AGACACAGGCAACGGA	product="hypothetical protein"			
	DR consensus	12	CTGAATATAACTTTGATGCC GTTAGGCGTTGAGCAC	*Leptospira interrogans* serovar Linhai str.56609"plasmid *lcp1*,	100	100	None

**Total CRISPR:16**	Spacer	8	ACAAAAGCAGCATATCTAG ACTTATCCAAAATAGAT	product="hypothetical protein"			
**1489**	DR consensus	4	GTGCTCAACGCCTAACGGCA TCAAAGTTATATTCAG	*Leptospira interrogans* serovar Hardjo-prajitno str. Hardjo	100	100	None
	Spacer	3	TCTTCCGAAACGGAAACAC GCCAAGGGTCTATAATTT	product="plasmid replication initiation factor"			
	DR consensus	4	GTGCTCAACGCCTAACGGCA TCAAAGTTATATTCAG	*Leptospira interrogans* serovar Linhai str.56609"plasmid *lcp1*,	100	100	None
	Spacer	8	AGGGGCGCTTTTCCGGGTCC TCATTGTTCTATTTT	product="hypothetical protein"			
	DR consensus	4	GTGCTCAACGCCTAACGGCA TCAAAGTTATATTCAG	*Leptospira interrogans* serovar Linhai str.56609"plasmid *lcp1*,	100	94.44	None
	Spacer	9	TTCCAGCATTGGCGGGGAAC CCTCAAGCATTGCAAT	product="hypothetical protein"			
	DR consensus	4	GTGCTCAACGCCTAACGGCA TCAAAGTTATATTCAG	*Leptospira interrogans* serovar Linhai str.56609"plasmid *lcp1*,	100	100	None
**Total CRISPR:15**	Spacer	12	GCGCTGTCGTTTGCGGGCAA GCCGTAAGACCGCAT	product="hypothetical protein"			
**1548**	DR consensus	8	CTGAATATAACTTTGATGCC GGTAGGCGTTGAGCAC	*Leptospira interrogans* serovar Linhai str.56609"plasmid *lcp3*,	100	97.37	None
**Total CRISPR:13**	Spacer	1	AGTTACTCAAAAATACTCAA TTAGAGGAGTATTGGGGA	product="hypothetical protein"			
